# An association between maternal weight change in the year before pregnancy and infant birth weight: ELFE, a French national birth cohort study

**DOI:** 10.1371/journal.pmed.1002871

**Published:** 2019-08-20

**Authors:** Marion Lecorguillé, Madalina Jacota, Blandine de Lauzon-Guillain, Anne Forhan, Marie Cheminat, Marie-Aline Charles, Barbara Heude

**Affiliations:** 1 Université de Paris, Centre of Research in Epidemiology and Statistics, INSERM, Institut national de la recherche agronomique, Paris, France; 2 APHP, Unité de Recherche Clinique, Hôpitaux Universitaires Paris île-de-France Ouest, Paris, France; 3 Institut national d’études démographiques, INSERM, Établissement français du sang, Joint Unit Elfe, Paris, France; Cornell University, UNITED STATES

## Abstract

**Background:**

Weight-control interventions in pregnant women with overweight or obesity have limited effectiveness for fetal growth and birth outcomes. Interventions or prevention programs aiming at the pre-pregnancy period should be considered. However, how the woman’s weight change before pregnancy affects fetal growth is not known. We investigated the association between weight change over the year before pregnancy and birth weight.

**Methods and findings:**

We used the inclusion data of 16,395 women from the ELFE French national birth cohort, a nationally representative cohort in which infants were enrolled at birth with their families in 2011. Maternal weight change was self-reported and classified into 3 groups: moderate weight variation or stable weight, weight loss > 5 kg, and weight gain > 5 kg or both weight loss and gain > 5 kg. Multiple linear regression models were used to investigate the association between pre-pregnancy weight change and a birth weight *z-*score calculated according to the French Audipog reference, adjusted for a large set of maternal characteristics. The analyses were stratified by maternal body mass index (BMI) at conception (<25 versus ≥25 kg/m^2^) and adjusted for BMI within these categories. We used the MacKinnon method to test the mediating effect of gestational weight gain (GWG) on these associations. Mother’s mean age was 30.5 years, 87% were born in France, and 26% had overweight or obesity. For women in either BMI category at conception, GWG was more than 2 kg higher, on average, for women with weight loss before pregnancy than for women with stable weight or moderate weight variation. For women with BMI < 25 kg/m^2^ at conception, birth weight was significantly higher with weight loss than stable weight before pregnancy (β = 0.08 [95% CI 0.02; 0.14], *p =* 0.01), and this total effect was explained by a significant mediating effect through GWG. For women with BMI ≥ 25 kg/m^2^ at conception, birth weight was not associated with pre-pregnancy weight loss during the year before pregnancy. Mediation analysis revealed that in these women, the direct effect of pre-pregnancy weight loss that would have resulted in a smaller birth weight *z-*score (β = −0.11 [95% CI −0.19; −0.03], *p =* 0.01) was cancelled out by the GWG. The mediating effect of GWG was even higher when weight loss resulted from a restrictive diet in the year before pregnancy. Weight gain before pregnancy was not associated with birth weight. Although we included a large number of women and had extensive data, the only potential cause of pre-pregnancy weight loss that was investigated was dieting for intentional weight loss. We have no information on other potential causes but did however exclude women with a history of pre-pregnancy chronic disease. Another limitation is declaration bias due to self-reported data.

**Conclusions:**

Health professionals should be aware that GWG may offset the expected effect of weight loss before conception on fetal growth in overweight and obese women. Further studies are required to understand the underlying mechanisms in order to develop weight-control interventions and improve maternal periconceptional health and developmental conditions for the fetus.

## Abstract 2

Marion Lecorguillé and colleagues reveal reduced birthweight in infants where obese mothers reduce weight prior to pregnancy, but these women are more likely to gain weight during pregnancy than stable weight women.

## Author summary

**Why was this study done?:**

- Maternal obesity is associated with macrosomia at birth and long-term health consequences for the offspring.
- Results from weight-control interventions during pregnancy suggest that it is too late to address obesity consequences once the pregnancy has already started and that more attention should be paid to the preconception period.
- The impact of maternal weight changes before pregnancy on infant birth weight has not been thoroughly investigated.

**What did the researchers do and find?:**

- We assessed the association between maternal reported weight changes during the year before pregnancy and infant birth weight in the ELFE French national birth cohort.
- For women with overweight and obesity, weight loss before pregnancy was associated with increased gestational weight gain compared to women with stable weight before pregnancy, which seemed to cancel out an expected reduction of infant birth weight.

**What do these findings mean?:**

- For women with overweight and obesity, weight-control interventions before pregnancy could be beneficial for fetal growth. Health professionals should be aware of a potential weight gain rebound during pregnancy after a weight loss before pregnancy.
- Further studies are required to understand the role of weight changes in the preconception period in order to refine nutritional prevention messages for women of childbearing age.

## Introduction

Reducing adverse pregnancy and fetal outcomes for women with overweight and obesity is a public health priority. Maternal obesity is a risk factor for maternal complications during pregnancy and for infants being large for gestational age (LGA) [1–3]. Also, maternal obesity during pregnancy has been associated with long-term health consequences for the offspring, such as increased body mass index (BMI) during infancy, childhood, and later life and increased risk of type 2 diabetes in adulthood [4,5]. Excessive gestational weight gain (GWG) can also contribute to increased risk of poor maternal and birth outcomes [1,6]. The Institute of Medicine recommends GWG ranges during pregnancy, according to pre-pregnancy BMI category, that are associated with good maternal and infant outcomes [7,8].

Some interventions to prevent or reduce obesity and its consequences have been implemented during the pregnancy period [9]. Lifestyle interventions during pregnancy could reduce GWG [10]. However, further studies have suggested that for women with overweight and obesity, diet and lifestyle interventions during pregnancy have very limited impact on other pregnancy outcomes, birth weight, and overweight risk in offspring [11–13]. These results are consistent with those from observational studies showing that high BMI before pregnancy was a stronger predictor of the risk of LGA than was excessive GWG [14]. Altogether, these results suggest that it is too late to address obesity consequences once the pregnancy has already started and that more attention should be paid to the preconception period.

The periconception period may represent a critical window during which nutritional exposure can influence embryo development and risk of obesity in the offspring. Similar maternal weight status at the start of pregnancy may result from distinct weight trajectories before pregnancy, which reflect particular nutritional and metabolic states. Different dynamics in preconception weight could specifically influence fetal growth and play a distinct role from the effect of nutritional stores during pregnancy. However, few studies have evaluated the association of maternal weight changes before pregnancy and fetal growth. Most human studies on this topic have addressed the impact of inter-pregnancy weight changes [15–17]. Epidemiological data have shown that in obese women, weight gain before pregnancy is associated with an increased risk of complications during pregnancy and macrosomia at birth [15–19]. Further studies have suggested decreased risk of LGA with weight loss between 2 pregnancies or after bariatric surgery for obese women [15,16,20]. However, increased risk of small for gestational age (SGA) has also been observed in association with bariatric surgery before pregnancy [21,22]. Weight loss before pregnancy could help reduce pregnancy and perinatal complications in overweight and obese women, but studies are needed to ensure that it has no harmful side effects. Conversely, women who were underweight at conception showed increased risk of preterm delivery; another study showed, for those who lost weight before pregnancy, a risk of fetal growth restriction and low infant birth weight [23,24]. Hence, the consequences for pregnancy outcomes of weight change before pregnancy may differ according to maternal BMI status at conception.

### Objective

This study aimed to investigate the association between maternal weight variation in the year before pregnancy and birth weight in a national birth cohort study in France. We hypothesized that maternal weight variation before pregnancy could be involved in the mechanisms programming fetal growth. Weight loss before pregnancy could be clinically relevant in overweight and obese women but not in normal-weight women, so we a priori stratified our analysis according to weight status at the beginning of pregnancy.

## Methods

This study is reported as per the Strengthening the Reporting of Observational Studies in Epidemiology (STROBE) guideline (S1 STROBE guideline checklist). A brief analysis plan was written and approved before starting statistical analyses (S1 Protocol).

### Study

The ELFE study (Étude Longitudinale Française depuis l’Enfance) is a French national longitudinal birth cohort with more than 18,000 children included at birth. The rationale and design of the ELFE cohort were previously detailed [25]. Recruitment took place on 25 selected days during 4 periods in 2011. The inclusion criteria were birth at 33 weeks’ amenorrhea or more, singleton or twin birth, and mother > 18 years, who gave informed consent and did not plan to leave metropolitan France within 3 years. Participation in the cohort was proposed to women who gave birth in 349 maternity hospitals randomly selected among the 544 public and private maternity hospitals in metropolitan France. Among eligible mothers, 51% agreed to participate (*N* = 18,040). The ELFE study was approved by an ethics committee (Comité de Protection des Personnes), the national committee on information concerning health research (Comité Consultatif sur le Traitement de l’Information en Matière de Recherche dans le domaine de la Santé), and the data protection authority (Commission Nationale de l’Informatique et des Libertés).

### Data collection

We used data collected from the following sources in the maternity wards after birth: medical records (S1 Questionnaire), face-to-face interviews (S2 Questionnaire), and self-administered questionnaires (S3 Questionnaire). A telephone questionnaire answered by the parents at 2 months after birth was also used to complete information on sociodemographic characteristics.

#### Preconception weight variations

The maternal self-administered questionnaire collected information on preconception weight variations during the year before pregnancy, with 5 possible answers: gain of >5 kg, gain of 2 to 5 kg, stable, loss of 2 to 5 kg, and loss of > 5 kg. We classified weight variation into 3 groups: weight loss > 5 kg; stable weight or moderate variation (absolute weight change ≤ 5 kg) (subsequently termed “stable weight”); and weight gain > 5 kg or both a loss > 5 kg and gain > 5 kg (subsequently termed “weight gain”). We grouped stable weight and moderate variation (absolute weight change ≤ 5 kg) in a single “stable weight” category because birth weight was not significantly different between the different groups. Some women indicated both a weight gain > 5 kg and a loss > 5 kg in the year before pregnancy. Because these women were too few (*N =* 191) to be considered a separate group, they were grouped with the “weight gain” category.

The women also declared whether they had followed a restrictive diet to lose weight during the year before pregnancy.

#### Fetal growth and anthropometric measures

We collected gestational age, child sex, and anthropometry at birth from medical records. *z*-Scores for birth weight were calculated according to French Audipog reference [26] taking into account gestational age and sex at birth. We also wanted to give a clinical interpretation of the results and investigated birth weight *z-*score in the categories SGA (<10th percentile), appropriate for gestational age (10th to 90th percentile), and LGA (>90th percentile).

#### Maternal variables

Sociodemographic data collected included maternal age (continuous), parity, education level (lower secondary, upper secondary, post-secondary, or tertiary education) according to the International Standard Classification of Education [27], activity status at the beginning of pregnancy (staying at home [including housewife, parental leave, unemployment] versus employed or student), living with a partner (yes versus no), place of birth (born in France versus other country), and pregnancy caregiver (gynecologist, midwife, general practitioner or none, or multiple professionals).

Health-related variables collected included health insurance coverage (regular versus related to precarious situations), smoking before and during pregnancy (yes versus no), and GWG (continuous). Pre-pregnancy BMI was calculated as weight (kg) divided by height squared (m²) and classified into 4 categories according to World Health Organization thresholds: underweight, <18.5 kg/m^2^; normal weight, 18.5 to <25.0 kg/m^2^; overweight, 25.0 to <30.0 kg/m^2^; and obesity, ≥30.0 kg/m^2^. Maternal GWG was calculated as measured weight at the end of pregnancy minus weight before pregnancy as reported by mothers.

Preexisting type 1 or 2 diabetes or gestational diabetes and medical history of chronic hypertension or gestational hypertension were retrieved from medical records. In addition, medical history of chronic severe disease or disabilities recorded in the medical record was collected by the research assistant and coded according to the International Classification of Diseases, 10th revision.

### Population selection

**Fig 1:**
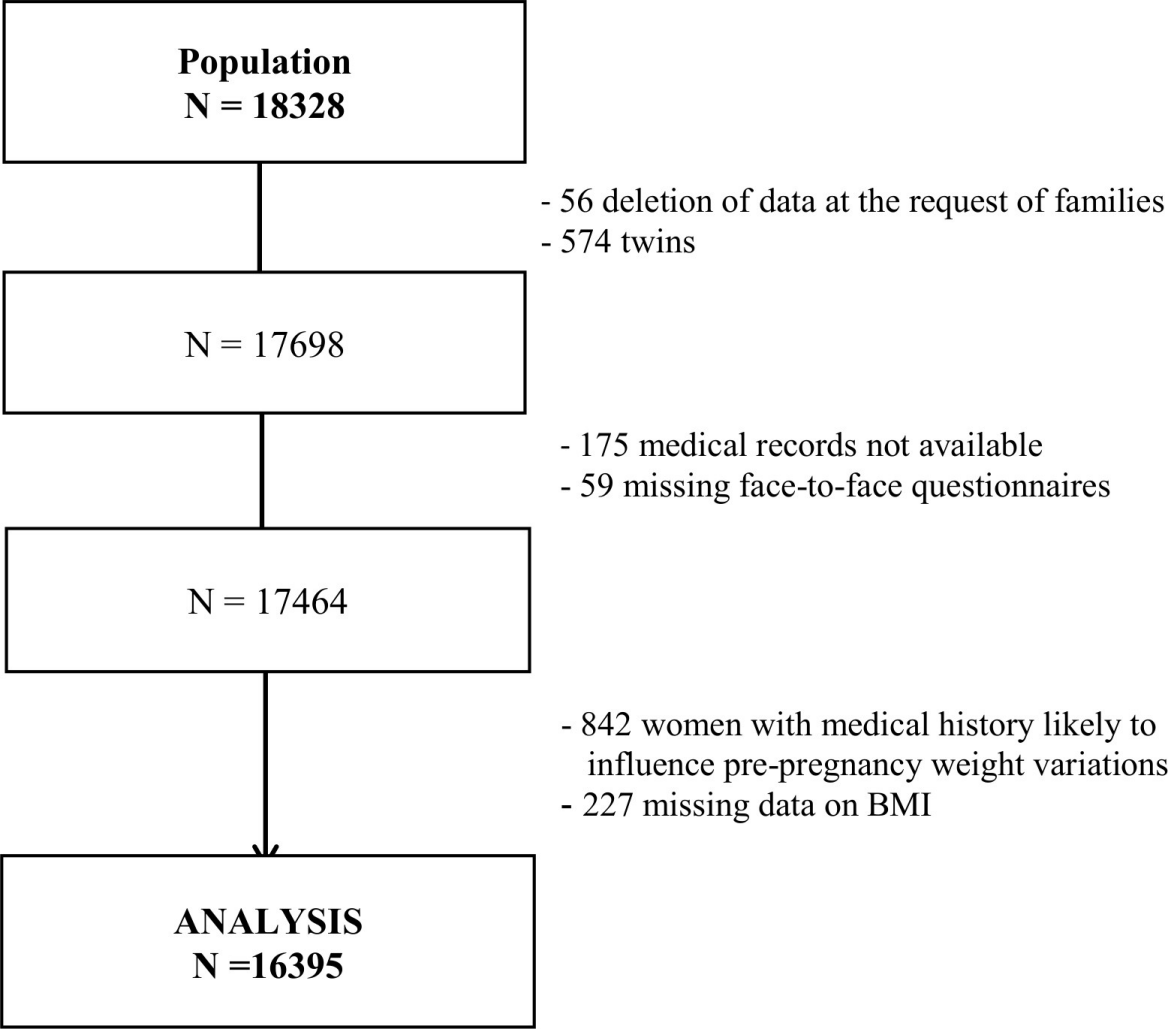
Selection of women for analysis.

Among the 18,040 mothers, who gave birth to 18,328 infants included in the study, 56 withdrew from the study and asked for data deletion. We excluded 574 twins from the analyses, as well as 175 and 59 mothers with missing medical records and face-to-face interviews, respectively. Finally, we also excluded 842 women with a medical history before pregnancy that may result in weight changes (history of metabolic or endocrine disease, thyroid disease, autoimmune disease, depression, psychological disorder such as anorexia, epilepsy, digestive disease, Crohn disease, bariatric surgery, infectious disease, cancer or congenital anomaly, chronic hypertension, or type 1 or 2 diabetes). Finally, 227 records with missing data on BMI, a key stratification variable, were not considered in the analysis. Fig 1 summarizes the steps of the population selection, which resulted in data for 16,395 women included in the analysis.

### Missing data imputation process

Most variables had less than 3% missing data, except for weight changes before pregnancy (collected by the self-administered questionnaire), which had about 13% missing data. Missing data for exposures, outcomes, and confounders were imputed using the SAS “MI” procedure. We compared women with and without a completed self-administered questionnaire and included all variables with significant difference in the imputation process (sociodemographic factors, birth characteristics, and maternal medical history). We generated 5 imputed datasets using the fully conditional specification method (S1 Table). The results of different imputed datasets were combined using the SAS “MI analyse” procedure, and standard errors were calculated using Rubin’s rules, which take into account the variability between the multiple regressions in imputed datasets [28,29].

### Statistical analysis

Characteristics of mothers and their children are described with mean ± SD and frequency (%) before imputation. We compared sociodemographic characteristics between women with different weight variations before pregnancy by chi-squared test for categorical variables and ANOVA for continuous variables, before imputation. Linear regression analyses were used to investigate the association between weight changes in the year before pregnancy and birth weight *z-*score (hereafter called “birth weight”). Logistic regression was used for analyzing the risk of SGA (SGA/no SGA) and LGA (LGA/no LGA). Women in the weight loss or gain group were compared to women with stable weight. We adjusted our analysis for the following confounders after careful selection based on directed acyclic graphs [30]: level of education, maternal age, smoking before and during pregnancy, place of birth, parity, health insurance coverage, and activity status. We stratified our analysis on weight status (i.e., maternal BMI at conception: <25 versus ≥25 kg/m^2^) for clinical considerations because weight loss before pregnancy could be advised and beneficial in overweight and obese women but not in normal-weight or underweight women. The interaction between weight variation before pregnancy and weight status on birth weight was significant (complete-case analysis, *p =* 0.003). We additionally adjusted for BMI before pregnancy as a continuous variable within the 2 BMI categories. All analyses were performed with SAS version 9.3. *p <* 0.05 was considered statistically significant.

#### Mediation analysis

**Fig 2:**
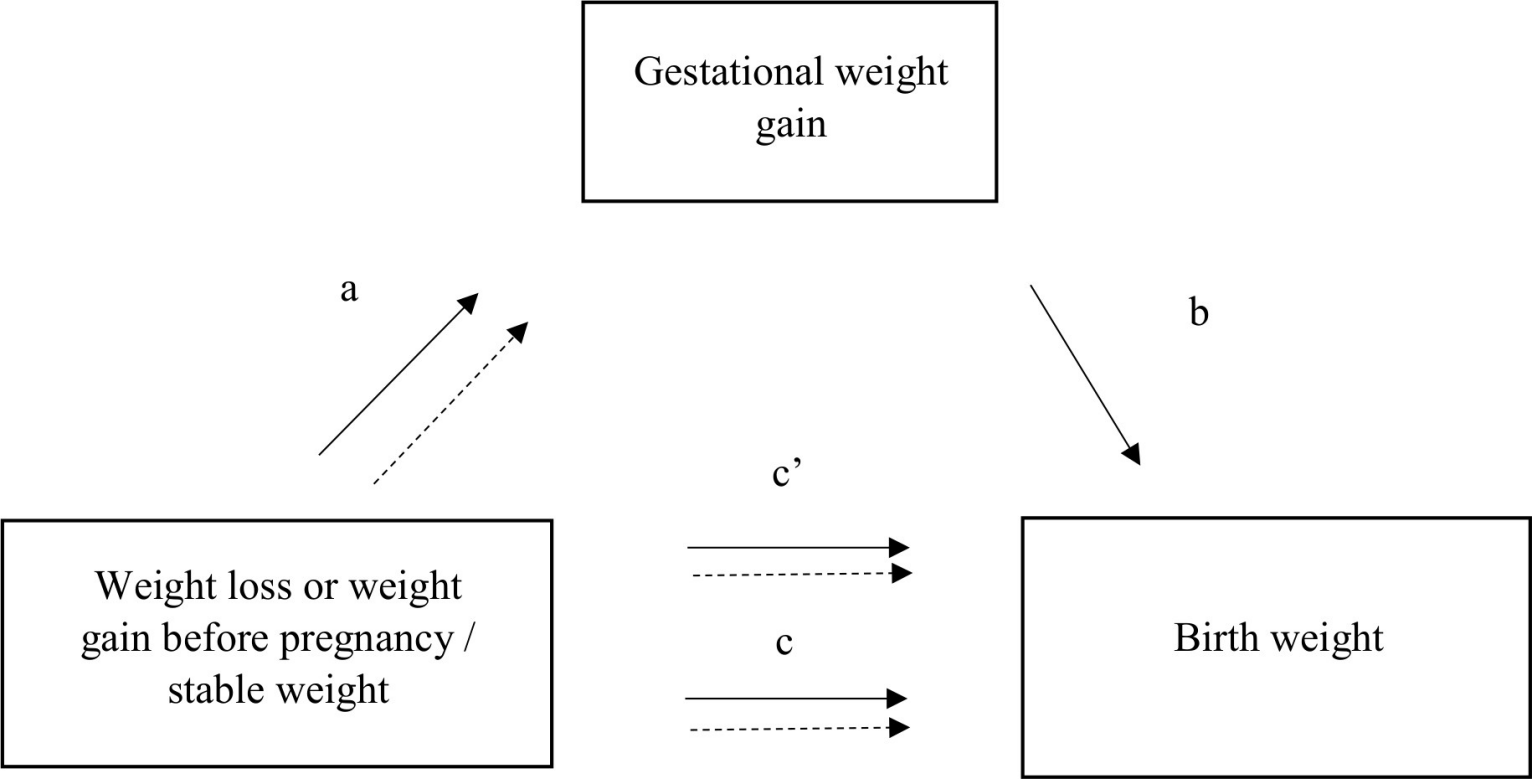
Mediator model. *a* is the association between weight variation and the potential mediator, gestational weight gain. *b* is the association between the potential mediator and the outcome variable, birth weight, adjusted for weight variation before pregnancy. *a* × *b* is the indirect effect. *c* is the total effect: overall association between weight variation and the outcome variable. *c*′ is the direct effect (non-mediated effect) adjusted for the mediator variable.

To address our mediation hypothesis, we used the method developed by MacKinnon et al. [31,32] (Fig 2). We hypothesized that GWG, which was associated with both the exposure (weight variation before pregnancy) and the dependent variable (birth weight), could be on the causal pathway. Indeed, we previously reported in the ELFE cohort that weight loss before pregnancy was associated with increased average weight gain during pregnancy whatever the BMI category before pregnancy (path *a* in Fig 2) [33]. Mediation analyses were stratified by the BMI at conception categories <25 and ≥25 kg/m^2^. The association between weight change (independent variable) and birth weight (dependent variable) adjusted for level of education, maternal age, smoking before and during pregnancy, place of birth, parity, health insurance coverage, activity status, and BMI corresponds to the total effect (path *c* in Fig 2). The direct effect, or non-mediated effect (path *c*′), is the effect of preconception weight change on birth weight via causal pathways other than influence on GWG, while the indirect effect is the effect that operates via the effect on GWG. The coefficient *b* relates the mediator to the dependent variable adjusted for the independent variable.

The product *a* × *b* corresponds to the mediation, or indirect, effect, which we tested by the significance of the Sobel test. For 95% standard normal confidence limits of the indirect effect, a critical value of 1.96 was used for the standard error [34].

#### Complementary analysis

To reinforce the validity of our results, we performed several additional analyses. We also stratified our analyses by restricting the group of women who lost weight before pregnancy to (1) those who declared dieting before pregnancy or (2) those who lost weight for another unspecified reason (not related to severe illness). We hypothesized that women who lost weight secondary to intentional restriction of food intake would be more exposed to weight regain after the restriction cessation that generally occurs with the start of pregnancy.

Another complementary analysis consisted of excluding women who gave birth to a child within the 2 years preceding the studied pregnancy. Indeed, in this subgroup, women experiencing physiological weight changes related to the previous pregnancy might have been classified, depending on the date of pregnancy, in the weight gain or weight loss category in the year before pregnancy.

Another concern is that GWG includes the baby’s weight. Therefore, the mediating effect of GWG could be overestimated, which would influence our estimate of the direct effect of weight change before pregnancy on birth weight. To explore this possibility, we studied, in a complementary analysis, the mediating effect of a variable called “maternal GWG,” calculated by subtracting child’s birth weight from GWG. Because the amount of GWG depends also on pregnancy duration, we performed a sensitivity analysis excluding premature birth.

Smoking behavior before and during pregnancy could modify the effect of weight variation before pregnancy on birth weight because many women could use smoking to restrict their appetite or weight gain before pregnancy. Therefore, we performed a complementary analysis restricted to women who did not smoke before and during pregnancy.

Finally, we re-ran all the analyses restricted to complete cases without missing data for exposure, outcome, and covariates.

## Results

### Population characteristics

The characteristics of the 16,395 women included in the analysis are summarized in Table 1 for the whole sample and by weight status, before multiple imputation of missing data. The women were mainly born in France (87%) and were employed or students (81%). More than 42% smoked before pregnancy, and 26% were overweight or obese.

**Table 1:** Description of the ELFE population (*N =* 16,395) after excluding women with a medical history or missing data on body mass index (BMI), in the whole sample and by BMI at the beginning of pregnancy.

Characteristic	Missing data *N* (%)	Population (*N* = 16,395)	BMI < 25 kg/m^2^ (*N =* 12,058)	BMI ≥ 25 kg/m^2^ (*N =* 4,337)
***Mother***				
**Maternal age (years), *n* (%)**	4 (0.02)			
<25		2,225 (13.6)	1,582 (13.1)	643 (14.8)
26–35		11,085 (67.6)	8,273 (68.6)	2,812 (64.8)
>35		3,081 (18.8)	2,199 (18.2)	882 (20.3)
**Born in France, *n* (%)**	12 (0.07)	14,272 (87.1)	10,601 (88.0)	3,671 (84.8)
**Maternal education, *n* (%)**	1 (0.01)			
Lower secondary		1,368 (8.3)	847 (7.0)	521 (12.0)
Upper secondary		5,711 (34.8)	3,841 (31.9)	1,870 (43.1)
Post-secondary		3,663 (22.3)	2,675 (22.2)	988 (22.8)
Tertiary		5,652 (34.5)	4,694 (38.9)	958 (22.1)
**Activity status, *n* (%)**	351 (2.1)			
Employed or student		12,935 (80.6)	9,839 (83.2)	3,096 (73.3)
Staying at home		3,109 (19.4)	1,982 (16.8)	1,127 (26.7)
**Health insurance coverage, *n* (%)**	55 (0.3)			
For precarious situations		1,343 (8.2)	853 (7.1)	490 (11.4)
Regular		14,997 (91.8)	11,170 (92.9)	3,827 (88.6)
**Living with a partner, *n* (%)**	73 (0.4)	15,450 (94.7)	11,409 (95.1)	4,041 (93.5)
**Smoking before pregnancy, *n* (%)**	49 (0.3)	6,964 (42.6)	5,211 (43.3)	1,753 (40.6)
***Pregnancy***				
**Primiparous, *n* (%)**	31 (0.2)	7,313 (44.7)	5,680 (47.2)	1,633 (37.7)
**Pregnancy caregiver, *n* (%)**	112 (0.7)			
Gynecologist		10,718 (65.8)	7,958 (66.4)	2,760 (64.1)
Midwife		2,034 (12.5)	1,455 (12.1)	579 (13.4)
General practitioner or none		907 (5.6)	638 (5.3)	269 (6.2)
Multiple professionals		2,624 (16.1)	1,926 (16.1)	698 (16.2)
**Smoking during pregnancy, *n* (%)**	104 (0.6)	3,281 (20.1)	2,446 (20.4)	835 (19.4)
**Weight variation in the year before pregnancy, *n* (%)**	2,102 (12.8)			
Weight loss		1,415 (9.9)	806 (7.7)	609 (16.2)
Stable weight		11,210 (78.4)	8,952 (85.0)	2,258 (60.0)
Weight gain		1,668 (11.7)	771 (7.3)	897 (23.8)
**Restrictive diet before pregnancy, *n* (%)**	1,974 (12.0)	2,381 (16.5)	1,202 (11.3)	1,179 (31.1)
**Pre-pregnancy BMI category**	0 (0)			
Underweight		1,292 (7.9)	1,292 (10.7)	—
Normal		10,766 (65.7)	10,766 (89.3)	—
Overweight		2,791 (17.0)	—	2,791 (64.4)
Obesity		1,546 (9.4)	—	1,546 (35.6)
**Gestational weight gain (kg), mean ± SD**	114 (0.7)	13.2 ± 5.5	13.9 ± 4.8	11.2 ± 7
***Newborn***				
**Gestational age of birth (WA), mean ± SD**	111 (0.7)	39.6 ± 1.4	39.6 ± 1.4	39.7 ± 1.4
**Birth weight Audipog *z*-score**^a^**, mean ± SD**	322 (2.0)	0.08 ± 1.0	0.01 ± 0.9	0.27 ± 1.0
**Birth weight category**^b^**, *n* (%)**	322 (2.0)			
Small for gestational age		1,280 (8.0)	1,012 (8.6)	268 (6.3)
Appropriate for gestational age		13,238 (82.4)	9,866 (83.5)	3,372 (79.2)
Large for gestational age		1,555 (9.7)	939 (7.9)	616 (14.5)

^a^Birth weight *z-*score according to the French Audipog reference [26].

^b^Birth weight categories to assess fetal growth: small for gestational age (<10th percentile), appropriate for gestational age (10th to 90th percentile), and large for gestational age (>90th percentile) according to the French Audipog reference.

WA, weeks’ amenorrhea.

### Statistical analysis

#### Univariate analysis

Women with weight loss before pregnancy more frequently stayed at home (20%) as compared with women with stable weight. We observed a significant difference between the 3 groups of weight variation before pregnancy in terms of smoking behavior (*p <* 0.01). Women who lost weight before pregnancy were more often smokers before pregnancy, and the proportion of these women who stopped smoking during pregnancy was about 26% and was greater than that for women with stable weight or weight gain before pregnancy. Results were similar whatever the weight status at conception. Overall, 57% of women with weight loss before pregnancy declared following a restrictive diet during the year before pregnancy (S2 Table).

#### Comparison of women by weight trajectory before pregnancy

Consistent with what was reported in a previous study of the ELFE cohort [33], weight loss and weight gain before pregnancy were associated with increased GWG after adjustment for confounders and BMI at conception. For women with BMI < 25 kg/m^2^ at conception, those with weight loss before pregnancy gained more than 2 additional kilograms during pregnancy (β = 2.20 [95% CI 1.79; 2.61], *p <* 0.01) than women with stable weight before pregnancy (S3 Table). Weight gain during pregnancy was also higher among women with weight gain before pregnancy than those with stable weight (β = 1.12 [95% CI 0.79; 1.45], *p <* 0.01). For women with BMI ≥ 25 kg/m^2^ at conception, those with weight loss before pregnancy gained about 2.8 kg more during pregnancy (β = 2.76 [95% CI 2.21; 3.32], *p <* 0.01) than women with stable weight before pregnancy. Also, women with weight gain before pregnancy had a persistent dynamic of excessive weight gain during pregnancy, resulting in >1.6 kg higher GWG (β = 1.61 [95% CI 1.12; 2.11], *p <* 0.01) compared with women with stable weight.

#### Association with birth weight *z-*score

For women with pre-pregnancy BMI < 25 kg/m^2^, in multivariable models adjusted in particular for pre-pregnancy BMI, the difference in mean birth weight *z-*score was significantly increased for offspring of mothers with pre-pregnancy weight loss as compared with women with stable weight (β = 0.08 [95% CI 0.02; 0.14], *p =* 0.01) (Table 2). Pre-pregnancy weight loss was not significantly associated with the risk of SGA and LGA, although the risk of SGA was non-significantly decreased (β = 0.78 [95% CI 0.58; 1.05], *p =* 0.10). Among women with pre-pregnancy BMI ≥ 25 kg/m^2^, weight loss before pregnancy was not associated with birth weight for a given pre-pregnancy BMI. Offspring of mothers with pre-pregnancy weight gain had increased birth weight and risk of LGA, although not significantly (β = 1.19 [95% CI 0.94; 1.49], *p =* 0.15).

**Table 2:** Association between weight variation in the year before pregnancy and birth weight in unadjusted and adjusted model stratified by BMI (*N =* 16,395).

Weight change during the year before pregnancy	Birth weight *z-*score (Audipog), β (95% CI)		Small for gestational age^a^, OR (95% CI)		Large for gestational age^a^, OR (95% CI)	
	Unadjusted model	Adjusted model	Unadjusted model	Adjusted model	Unadjusted model	Adjusted model
**BMI < 25 kg/m**^**2**^ **(*N =* 12,058)**						
Weight loss^b^	**0.14 (0.08; 0.21)**	**0.08 (0.02; 0.14)**	**0.71 (0.53; 0.95)**	0.78 (0.58; 1.05)	1.15 (0.89; 1.47)	0.98 (0.75; 1.26)
Stable weight^b^	0 (Ref)	0 (Ref)	0 (Ref)	0 (Ref)	0 (Ref)	0 (Ref)
Weight gain^b^	0.04 (−0.04; 0.12)	0.02 (−0.06; 0.10)	1.01 (0.76; 1.33)	0.98 (0.74; 1.30)	1.16 (0.87; 1.54)	1.09 (0.81; 1.48)
**BMI ≥ 25 kg/m**^**2**^ **(*N =* 4,337)**						
Weight loss^c^	−0.01 (−0.09; 0.07)	−0.02 (−0.1; 0.06)	1.14 (0.80; 1.62)	1.13 (0.79; 1.61)	1.00 (0.77; 1.31)	0.96 (0.73; 1.26)
Stable weight^c^	0 (Ref)	0 (Ref)	0 (Ref)	0 (Ref)	0 (Ref)	0 (Ref)
Weight gain^c^	0.03 (−0.05; 0.11)	0.03 (−0.05; 0.11)	1.02 (0.73; 1.42)	0.96 (0.67; 1.37)	1.21 (0.97; 1.51)	1.19 (0.94; 1.49)

Adjusted model adjusted for maternal education level, maternal age, smoking before and during pregnancy, place of birth, parity, health insurance coverage, activity status, and pre-pregnancy BMI.

^a^Small for gestational age (<10th versus ≥10th percentile) and large for gestational age (>90th versus ≤90th percentile) according to the French Audipog reference [26].

^b^Minimum–maximum number of women in each category of weight variation before pregnancy depending on imputed tables: weight loss, 910–944; stable weight, 10,177–10,192; and weight gain, 923–956.

^c^Minimum–maximum number of women in each category of weight variation before pregnancy depending on imputed tables: weight loss, 690–703; stable weight, 2,543–2,564; and weight gain, 1,072–1,097.

### Mediation analysis

**Fig 3:**
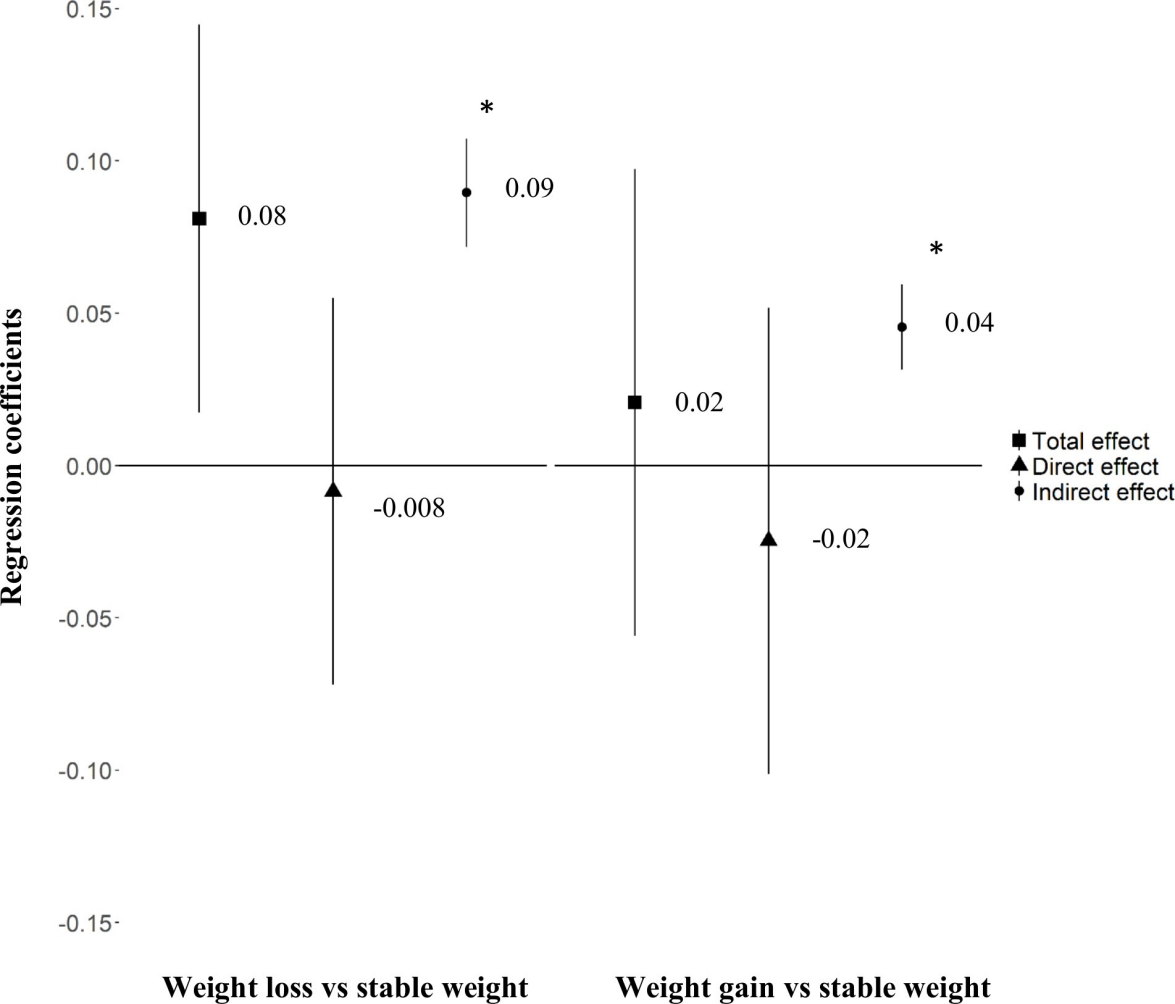
Association between weight variation before pregnancy and birth weight mediated by gestational weight gain for women with pre-pregnancy BMI < 25 kg/m^2^. Effect size β (95% CI). All models were adjusted for maternal education level, maternal age, smoking before and during pregnancy, place of birth, parity, health insurance coverage, activity status, and pre-pregnancy BMI. Birth weight *z-*score according to the French Audipog reference [26]. *Sobel test of indirect effect *p* < 0.001.

For women with BMI < 25 kg/m^2^ before pregnancy, increased GWG predicted increased birth weight (β = 0.041 [95% CI 0.037; 0.044], *p <* 0.01). The positive association between weight loss before pregnancy and birth weight (total effect) for a given pre-pregnancy BMI was fully mediated by the indirect effect of GWG, the direct effect being close to 0 (β = −0.008 [95% CI −0.07; 0.05], *p =* 0.79) (Fig 3).

**Fig 4:**
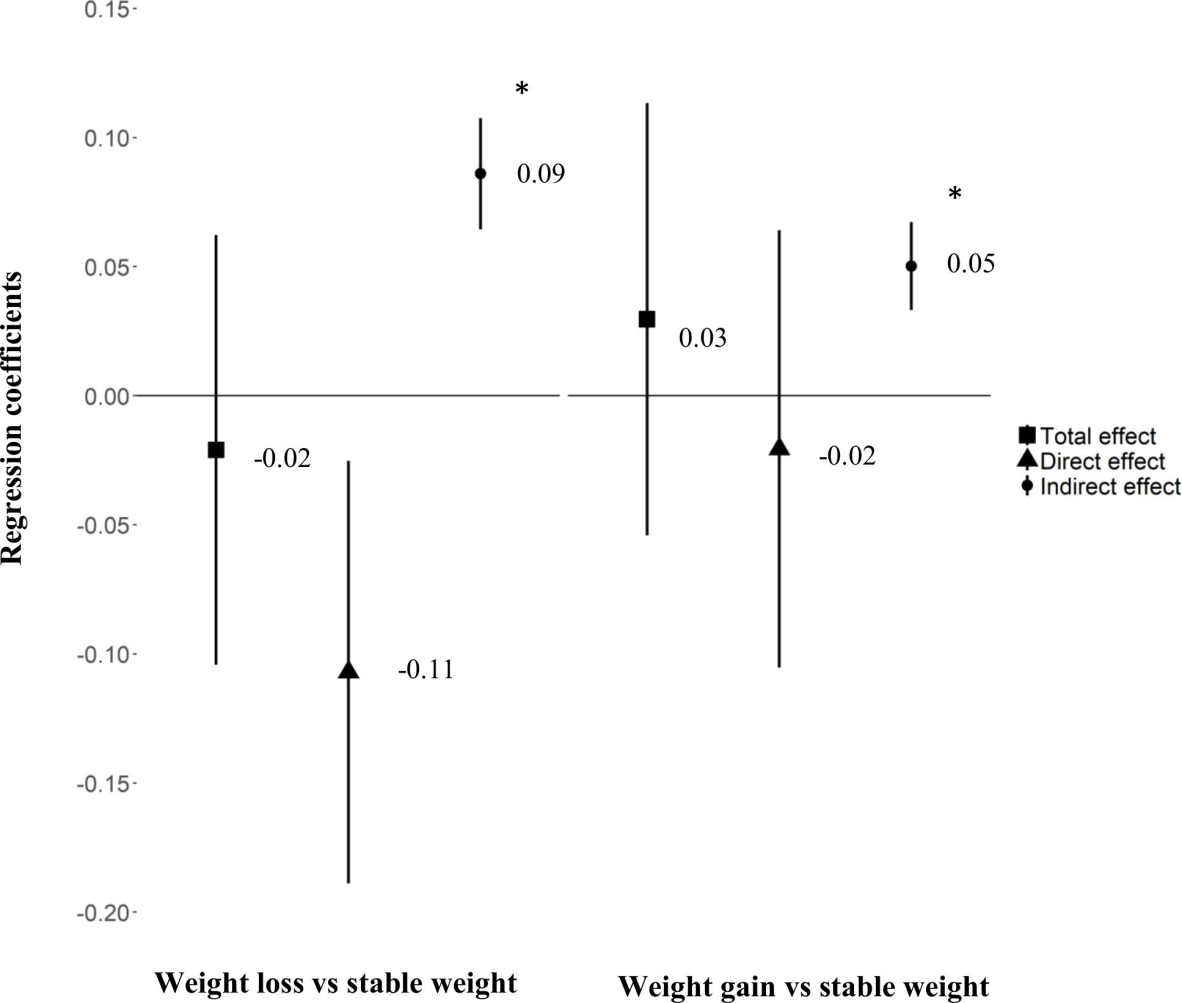
Association between weight variation before pregnancy and birth weight mediated by gestational weight gain for women with BMI ≥ 25 kg/m^2^. Effect size β (95%CI). All models were adjusted on maternal education level, maternal age, smoking before and during pregnancy, place of birth, parity, health insurance coverage, activity status, and pre-pregnancy BMI. Birth weight *z-*score according to the French Audipog reference [26]. *Sobel test of indirect effect *p* < 0.001.

For women with BMI ≥ 25 kg/m^2^, increased GWG was also associated with increased birth weight (β = 0.030 [95% CI 0.026; 0.036], *p <* 0.01). The association between pre-pregnancy weight loss and birth weight was not significant, but when the model was adjusted for GWG, weight loss before pregnancy had a significant negative direct effect on birth weight (β = −0.11 [95% CI −0.19; −0.03], *p =* 0.01) for a given pre-pregnancy BMI (Fig 4). We found no significant association between weight gain before pregnancy and birth weight in the 2 BMI groups. However, we observed a significant mediating effect of GWG.

The results were even stronger when we restricted the analysis to women with BMI ≥ 30 kg/m^2^ (*N =* 1,546), for a direct effect (β = −0.19 [95% CI −0.33; −0.06], *p <* 0.01) and negative total effect (β = −0.12 [95% CI −0.26; 0.014], *p =* 0.07). The direct effect was lower for overweight than obese women (β = −0.06 [95% CI −0.17; 0.04], *p =* 0.24), but we found a persistent and higher effect of GWG in this group (β = 0.10 [95% CI 0.07; 0.13], *p <* 0.001).

### Complementary analyses

#### Restrictive diet

Among women with pre-pregnancy BMI ≥ 25 kg/m^2^, 1,179 had followed a restrictive diet in the year before pregnancy and 2,608 had not. The mediating effect of GWG was increased for women who had dieted and lost weight in the year before pregnancy (β = 0.09 [95% CI 0.05; 0.12], *p <* 0.001) (S1 Fig).

#### Exclusion of women with a child born within 2 years before the current birth

In a complementary analysis, we excluded women who gave birth to a child within 2 years before the current birth (*N =* 2,925) to assess how much our results were driven by weight changes associated with a recent pregnancy. The results were globally unchanged: Both weight loss and weight gain were associated with increased subsequent GWG in the 2 BMI groups. For women with BMI ≥ 25 kg/m^2^, mediation analysis showed a negative direct effect of weight loss before pregnancy on birth weight, although of lower magnitude than in the whole sample (β = −0.07 [95% CI −0.17; 0.02], *p* = 0.11).

#### Analysis replacing GWG with our estimate of maternal GWG

In the complementary analysis for women with BMI ≥ 25 kg/m^2^ and weight loss before pregnancy, the mediating effect of maternal GWG was, as expected, reduced as compared with the main analysis (β = 0.06 [95% CI 0.04; 0.08], *p <* 0.01), but the mediation analyses still found a negative direct effect of pre-pregnancy weight loss only slightly lower than that in the main analysis (β = −0.08 [95% CI −0.16; 0.003], *p =* 0.06) (S2 Fig).

#### Sensitivity analysis

In our analysis restricted to non-smoking women with BMI < 25 kg/m^2^ (*N =* 6,744), we found no association (total effect) between weight loss before pregnancy and birth weight (β = 0.02 [95% CI −0.07; 0.11]). For those with BMI ≥ 25 kg/m^2^ (*N =* 2,533), the direct effect of pre-pregnancy weight loss on birth weight was slightly lower than in the main analysis (β = −0.09 [95% CI −0.20; 0.03], *p =* 0.03, versus β = −0.11 [95% CI −0.19; 0.03]), but we still observed a significant and persistent indirect effect of GWG (β = 0.09 [95% CI 0.06; 0.12], *p <* 0.001).

When we excluded premature birth in our sensitivity analysis, our results were unchanged for both BMI categories.

#### Complete-case analysis

Similar results were obtained from analyses restricted to complete cases as for analyses with imputed data. We also tested the analyses in participants without imputation of the outcome (birth weight) but with imputation of covariates, and the results were also unchanged.

## Discussion

For women who were not overweight or obese at conception, we found a significant positive association between weight loss before pregnancy and birth weight that was totally mediated and explained by increased GWG. For women with overweight and obesity, we did not find an association between weight loss before pregnancy and birth weight. However, after taking into account GWG, weight loss before pregnancy had a negative direct effect on birth weight. GWG seemed to cancel out the expected effect on birth weight reduction of weight loss before pregnancy. For women with weight gain before pregnancy, GWG was increased during pregnancy and had a significant indirect effect on birth weight, but it did not translate into a significant total effect, and there was no direct association between weight gain before pregnancy and birth weight.

In contrast to our results, a previous French study evaluated weight change from age 20 years to pregnancy and showed that weight loss before pregnancy was associated with reduced fetal growth and with risk of SGA for women with BMI < 25 kg/m^2^ [24]. In this study, pre-pregnancy weight loss reflected a much longer period before pregnancy and less directly reflected the consequence of a recent weight loss, especially in relation to increased GWG. In addition, the analyses were adjusted for GWG, and therefore only tested a direct effect of the previous weight trajectory without considering GWG as a mediator. An American study including more than 10,000 obese women found an increased risk of macrosomia for women with inter-pregnancy weight retention, but weight loss (≥2 BMI units [kg/m^2^]) between pregnancies was associated with reduced risk of LGA. However, excessive weight loss (>8 BMI units) was related to increased SGA risk [16]. This finding highlights the need to also evaluate the risk of excessive weight loss before pregnancy for fetal development. Another study from a nationwide Swedish cohort also showed a dose-dependent increase in LGA risk with inter-pregnancy weight gain in overweight and obese women. The authors recommended weight loss after a pregnancy in overweight and obese women as well as prevention of weight gain before pregnancy for women with normal BMI [15]. After controlling for BMI at conception, we did not find an association between weight gain before pregnancy and birth weight. Women who self-reported weight gain in the year before conception showed a dynamic of excessive weight gain persisting over the pregnancy. However, their GWG was lower, on average, than that of women who lost weight before pregnancy. Obese women who gain weight before pregnancy are at risk of perinatal complications [15], and they might benefit from increased medical attention and monitoring during pregnancy. The differences between our study and results in the literature could also be explained by different time periods before pregnancy for considering weight change [35].

We have shown that a pre-pregnancy weight loss of >5 kg is associated with reduced birth weight, after taking into account GWG. For a given pre-pregnancy BMI, overweight and obese women with a decreasing weight trajectory may have a better metabolic health and lipid profile at the start of pregnancy [36,37]. Lower frequency of glucose intolerance, hyperinsulinemia, or lipidemia may explain our observed direct effect on decreased birth weight, because all of these factors contribute to fetal growth [38,39]. Moreover, animal studies have shown an association of weight loss and low-fat diet in obese animals with better offspring outcomes, such as reduced fat mass and improved metabolic and hepatic function in offspring [40–42].

However, weight loss is accompanied by several physiological changes such as altered storage of energy and modified nutritional status and hormone pathways involved in the regulation of appetite, which can predispose to weight regain [43]. GWG is a unique and complex mechanism that can be influenced especially by maternal metabolism and placental function [44]. Smoking cessation is also a well-known risk factor for weight gain. In our study, 26% of women who smoked before pregnancy stopped smoking during pregnancy. When we restricted our analyses to overweight and obese women with no history of smoking, our main result was similar: We observed a negative direct effect of weight loss before pregnancy on birth weight and a positive indirect effect of GWG.

These physiological conditions may facilitate the mechanisms of weight regain after caloric restriction. Other factors related to pregnancy such as a decrease in physical activity may also elicit weight regain.

The strengths of our study are the large number of women included and the availability of sociodemographic and medical data. We had information on the medical history of women and excluded women with diseases, psychological disorders, bariatric surgery, and metabolic status that could have affected weight change before pregnancy. The use of multiple imputation techniques limited bias due to missing data in variables of interest and confounders.

Among the limitations is the questionable accuracy of data reported by the women on their weight history before pregnancy. However, our analysis focused on large weight changes (± 5 kg) in a recent period (1 year before pregnancy). The accuracy would be similar for information collected by a heath professional at the start of pregnancy, and we feel that our results are relevant to a clinical situation. Another limitation is that our information on the potential cause of weight loss is limited to the practice of a restrictive diet, and information on the type of diet and dieting period in the year before pregnancy was lacking. We could not study whether our observed direct effect of weight loss on birth weight was due to the weight change itself or the modification of the maternal lifestyle driving it.

Using total GWG, including the baby’s weight, is also a study limitation. When adjusting for total GWG, we took out part of the association we were trying to assess between pre-pregnancy weight variation and birth weight. To circumvent this drawback, in a complementary analysis, we approached the maternal component of GWG by subtracting the child’s birth weight from the total GWG. We assumed that although GWG also includes placenta weight and amniotic fluid, the main source of variability of GWG minus birth weight was the maternal component. The results from this complementary analysis were similar to those of the main analysis, which supports our interpretation of the results.

Finally, we did not have information on intermediate weight measurements during pregnancy, which prevented us from assessing weight gain for specific periods or trimesters. GWG is not linear across gestation, and the rate of weight gain is slower in the first trimester [45]. Distinct kinetic GWG may have differential impact on fetal growth [46,47].

Only live births are included in the ELFE cohort. If weight change in the year before pregnancy affects the probability of miscarriage or stillbirth, our results could be biased, probably by underestimating the effect of extreme weight changes on fetal growth. A selection bias could also be possible if there were associations between, on the one hand, both fetal growth and weight change before pregnancy and, on the other, the probability of inclusion in the ELFE cohort. Except for low maternal education level, which was controlled for, we could not see any obvious reason why weight change before pregnancy would influence the probability of inclusion.

## Conclusion

Few studies have evaluated the association between pre-pregnancy weight variation and fetal development. Our results suggest that increased GWG after weight loss before pregnancy may obscure any beneficial effect on fetal growth. These results call for increased vigilance on GWG in women who lost weight or dieted before pregnancy.

## Supporting information

S1 Fig Association between weight loss before pregnancy and birth weight mediated by gestational weight gain for women with BMI ≥ 25 kg/m^2^ who had followed a restrictive diet or not. Effect size β (95% CI). All models were adjusted for maternal education level, maternal age, smoking before and during pregnancy, place of birth, parity, health insurance coverage, activity status, and pre-pregnancy BMI. Birth weight *z-*score according to the French Audipog reference [26]. *Sobel test of indirect effect *p* < 0.001. For women with BMI ≥ 25 kg/m^2^ before pregnancy, the minimum–maximum numbers of women with weight loss or stable weight before pregnancy depending on imputed tables are as follows: no restrictive diet—weight loss, 208–208; stable weight, 1,767–1,770; with restrictive diet—weight loss, 404–407; stable weight, 494–497.
(TIFF)

S2 Fig Association between weight variation before pregnancy and birth weight *z-*score mediated by gestational weight gain for women with a pre-pregnancy BMI ≥ 25 kg/m^2^. Sensitivity analysis removing birth weight from gestational weight gain (*N =* 4,337). Effect size β (95% CI). All models were adjusted for maternal education level, maternal age, smoking before and during pregnancy, place of birth, parity, health insurance coverage, activity status, and pre-pregnancy BMI. Birth weight *z-*score according to the French Audipog reference [26]. *Sobel test of indirect effect *p* < 0.001.
(TIFF)

S1 Protocol Description of the prospective analysis plan. (DOCX)

S1 Questionnaire Maternity medical record. (PDF)

S2 Questionnaire Maternity mother interview. (PDF)

S3 Questionnaire Maternal weight variation. (DOCX)

S1 STROBE guideline checklist (DOC)

S1 Table Details of the method used for multiple imputation. *Fully conditional specification method. **Percent interval of missing data for the different *z-*scores of fetal growth in the second and third trimester.
(DOCX)

S2 Table Characteristics of women by weight variation before pregnancy (*N =* 14,293) before imputation, after excluding women with a medical history and missing data for BMI. Data are percent (*n*) or mean ± SD. *By chi-squared test comparing the 3 groups of weight variation or ANOVA for continuous variables.
(DOCX)

S3 Table Association between weight variation before pregnancy and gestational weight gain by BMI category. Adjusted model: linear regression adjusted for maternal education level, maternal age, smoking before and during pregnancy, place of birth, parity, health insurance coverage, active status, and pre-pregnancy BMI. ^a^Minimum–maximum number of women in each category of weight variation before pregnancy depending on imputed tables: weight loss, 910–944; stable weight, 10,177–10,192; and weight gain, 923–956. ^b^Minimum–maximum number of women in each category of weight variation before pregnancy depending on imputed tables: weight loss, 690–703; stable weight, 2,543–2,564; and weight gain, 1,072–1,097.
(DOCX)

## Abbreviations

BMI: body mass index
GWG: gestational weight gain
LGA: large for gestational age
SGA: small for gestational age
