# Supplementary figures and images for: An association between maternal weight change in the year before pregnancy and infant birth weight: ELFE, a French national birth cohort study
Source: PLoS Med. 2019 Aug 20;16(8):e1002871. doi: 10.1371/journal.pmed.1002871 (PMC6701747; doi:10.1371/journal.pmed.1002871)

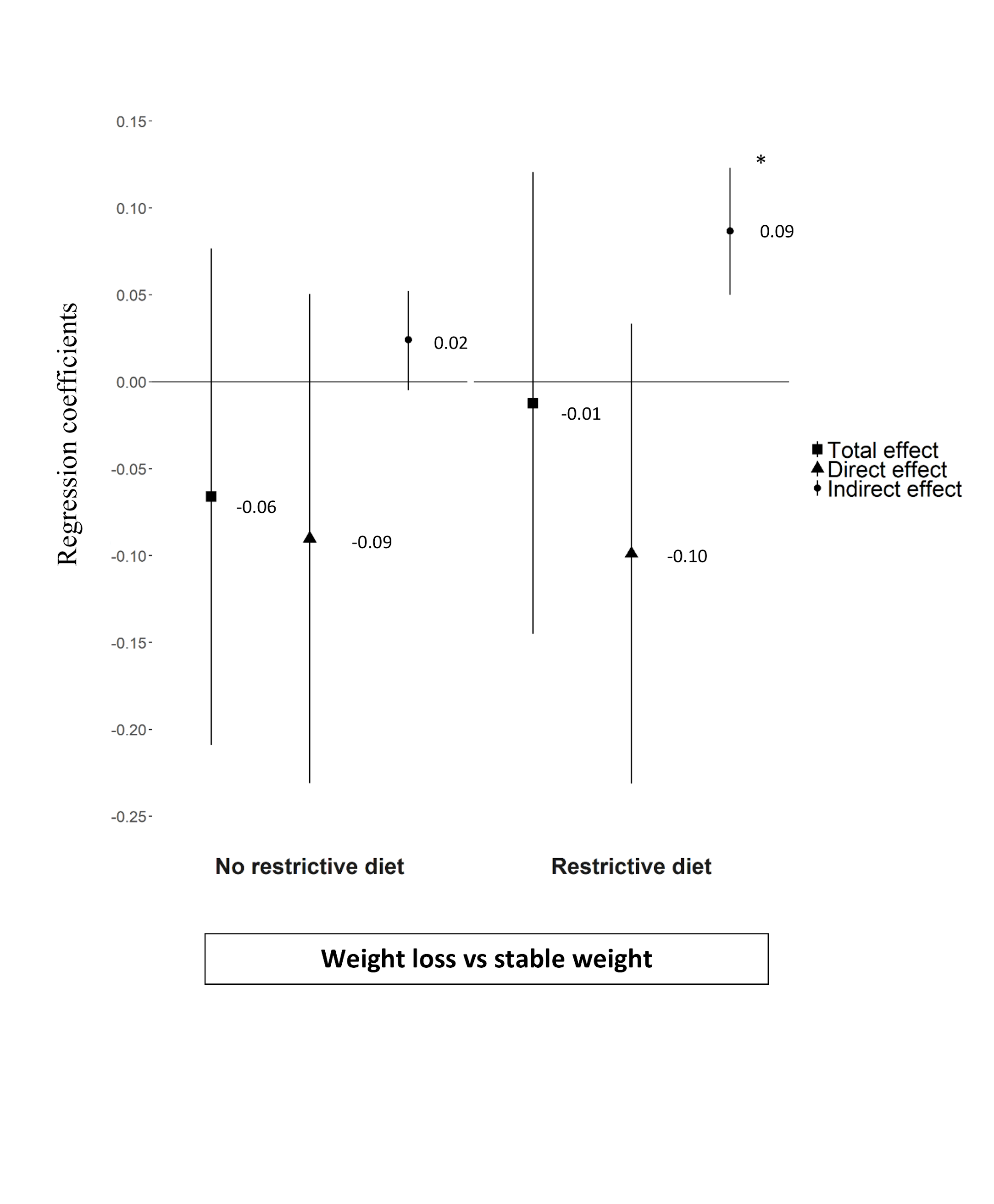

Supplement: S1 Fig — Effect size β (95% CI). All models were adjusted for maternal education level, maternal age, smoking before and during pregnancy, place of birth, parity, health insurance coverage, activity status, and pre-pregnancy BMI. Birth weight z-score according to the French Audipog reference [26]. *Sobel test of indirect effect p < 0.001. For women with BMI ≥ 25 kg/m2 before pregnancy, the minimum–maximum numbers of women with weight loss or stable weight before pregnancy depending on imputed tables are as follows: no restrictive diet—weight loss, 208–208; stable weight, 1,767–1,770; with restrictive diet—weight loss, 404–407; stable weight, 494–497. (TIFF) [file pmed.1002871.s001.tiff]

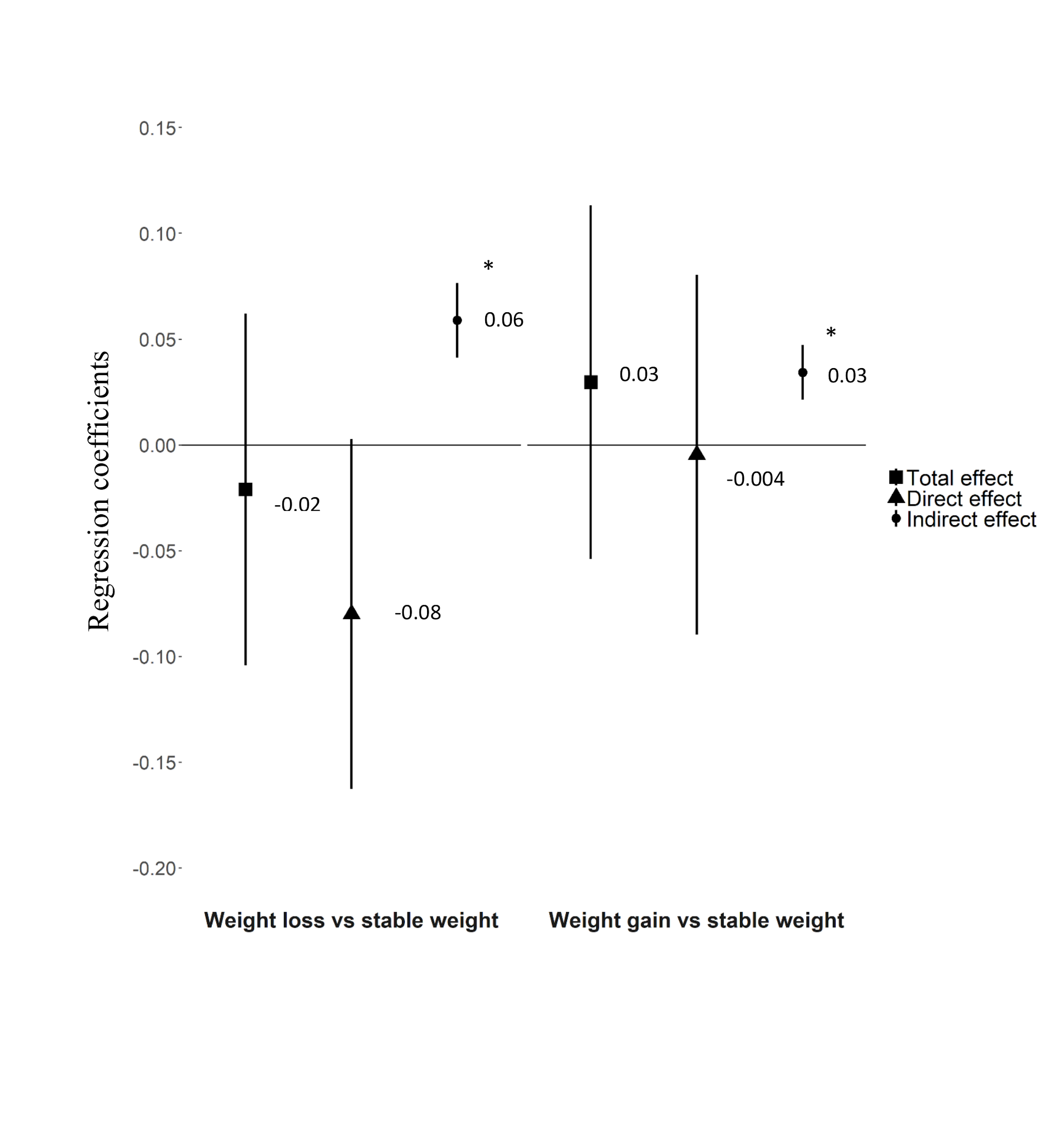

Supplement: S2 Fig — Sensitivity analysis removing birth weight from gestational weight gain (N = 4,337). Effect size β (95% CI). All models were adjusted for maternal education level, maternal age, smoking before and during pregnancy, place of birth, parity, health insurance coverage, activity status, and pre-pregnancy BMI. Birth weight z-score according to the French Audipog reference [26]. *Sobel test of indirect effect p < 0.001. (TIFF) [file pmed.1002871.s002.tiff]
