# Supplementary material for: An association between maternal weight change in the year before pregnancy and infant birth weight: ELFE, a French national birth cohort study
Source: PLoS Med. 2019 Aug 20;16(8):e1002871. doi: 10.1371/journal.pmed.1002871 (PMC6701747; doi:10.1371/journal.pmed.1002871)

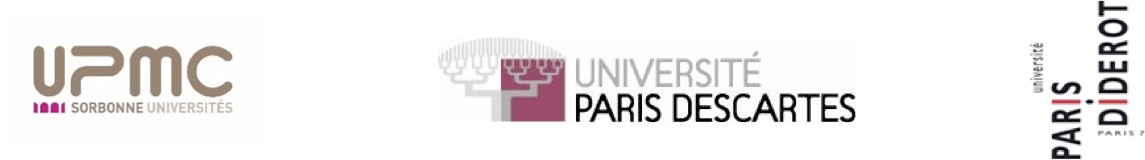


# PhD Subject

Laboratory: INSERM UMR 1153- Centre de Recherche Statistique Sorbonne Paris Cité (CRESS)

Team 6 EARoH: EArly life Research on later Health

PhD supervisor: Marie-Aline Charles

Co-director: Barbara Heude

Address: 16 avenue Paul Vaillant Couturier, Bâtiment Inserm 94807 Villejuif

**Maternal nutrition before and during pregnancy, epigenetics and fetal growth**

**I/ Introduction**

Increasing evidence suggests that early life exposure to environmental factors may influence long-term health. According to the Developmental Origins of Health and Disease (DOHAD) hypothesis, nutritional exposure during critical periods of development (such as pre-conceptional and gestational period) could affect development and risk of adverse effects on offspring health (1).

Many factors are important for a healthy pregnancy, such as body adipose tissue, reserve of nutrients and dietary intake. In France, many women may have sub-optimal nutritional status at the beginning of pregnancy. Among women who gave birth in 2011, the prevalence of obesity and thinness in early pregnancy was 10.8% and 7.8% respectively (2). Many studies have shown that the mother's body mass index (BMI) at conception and gestational weight gain are associated with birth weight (3). However, few studies have examined the maternal weight trajectory before pregnancy and their consequences on fetal growth (4). The periconception period may represent a critical window during which nutritional exposure can influence the embryo’s development and risk of obesity in the offspring (5). Similar maternal weight status at the start of pregnancy may result from distinct weight trajectories before pregnancy, which reflect particular nutritional and metabolic states. Epidemiologic data have shown that in obese women, weight gain before pregnancy is associated with increased risk of complications during pregnancy and macrosomia at birth (6-8). We reported in a previous study of the ELFE cohort that weight loss and weight gain before pregnancy were associated with increased GWG after adjustment for confounders and initial BMI.

**II/ Objectives**

This study aimed to investigate the association between maternal weight variation in the year before pregnancy and birth weight in the ELFE cohort. The hypothesis was that for the same BMI at the beginning of pregnancy, weight variation before pregnancy may be associated with fetal growth.

**III/ Materials and methods**

**a/ Data collection**

The ELFE cohort (Etude longitudinale Française depuis l'enfance) is the first French national longitudinal study that follows children and their families for 20 years to study how their environment affects their development, health and socialization from the intrauterine period to adolescence. Overall, 18329 children born in 2011 in one of the 349 metropolitan maternity hospitals in France were recruited for the ELFE study. The rationale and design of the ELFE cohort were previously detailed (9). Recruitment took place on 25 selected days during 4 periods in 2011. The inclusion criteria were birth at ≥ 33 weeks’ amenorrhea, singleton or twin births, mother’s age > 18 years, who gave informed consent and did not plan to leave metropolitan France within 3 years.

We will use collected information in the study: BMI at the beginning of pregnancy, weight variations before pregnancy, gestational weight gain, food frequency questionnaire related to the last 3 months during pregnancy, sociodemographic data, and medical data collected after birth.

For this study, a food intake adequacy score for the specific recommendations of pregnancy and adults was created (PNNS score) (2). The child's fetal growth will be studied both using anthropometric variables (weight, height and head circumference) collected at birth for the whole cohort and also ultrasonography data available for approximately 14,000 children.

**b/ Analysis Plan**

The first analysis will consist of studying the associations between weight variation categories or the restrictive diet before pregnancy and different variables characterizing fetal growth in the ELFE study. Weight loss before pregnancy could be clinically relevant in overweight women but not in normal-weight women, so we will a priori stratify our analysis by overweight status at the beginning of pregnancy. Linear regression analyses will be used to investigate the association between weight changes in the year before pregnancy and birth weight, adjusted for different confounders: level of education, maternal age, smoking before and during pregnancy, place of birth, parity, and BMI. Mediation analyses will be performed to assess the role of gestational weight gain in the relation.

**IV/ References**

1. Gluckman PD, Hanson MA, Cooper C, Thornburg KL. Effect of in utero and early-life conditions on adult health and disease. N Engl J Med. 2008;358:938-941.
2. Kadawathagedara M, Kersuzan C, Wagner S, Tichit C, Gojard S, Charles MA, et al. Adéquation des consommations alimentaires des femmes enceintes de l’étude ELFE aux recommandations du Programme national nutrition santé. Cah Nutr Diététique. 2017 Apr;52(2):78-88.
3. Yu Z, Han S, Zhu J, Sun X, Ji C, Guo X. Pre-Pregnancy Body Mass Index in Relation to Infant Birth Weight and Offspring Overweight/Obesity: A Systematic Review and Meta-Analysis. PLoS ONE. 2013 Apr 16;8(4):1-11.
4. Jacota M. Variations pondérales préconceptionelles et gestationnelles  : étude de leurs relations avec le diabète gestationnel et le développement de l’adiposité des enfants à 5-6 ans à partir des cohortes mère-enfant françaises EDEN et ELFE. Thèse d'épidémiologie. Université Paris-Sud;2016,172 p.
5. Zhang S, Rattanatray L, Morrison JL, Nicholas LM, Lie S, McMillen IC. Maternal obesity and the early origins of chilhood obesity: weighing up the benefits and costs of maternal weight loss in the periconceptionak period for the offspring. Exp Diabetes Res. 2011;2011:1-10.
6. Villamor E, Cnattingius S. Interpregnancy weight change and risk of adverse pregnancy outcomes: a population-based study. The Lancet. 2006;368: 1164–1170. doi:10.1016/S0140-6736(06)69473-7
7. Jain AP, Gavard JA, Rice JJ, Catanzaro RB, Artal R, Hopkins SA. The impact of interpregnancy weight change on birthweight in obese women. Am J Obstet Gynecol. 2013;208: 205.e1–7. doi:10.1016/j.ajog.2012.12.018
8. Getahun D, Ananth CV, Peltier MR, Salihu HM, Scorza WE. Changes in prepregnancy body mass index between the first and second pregnancies and risk of large-for-gestational-age birth. Am J Obstet Gynecol. 2007;196: 530.e1–8. doi:10.1016/j.ajog.2006.12.036
9. ELFE. Etude ELFE  : comment grandissent nos enfants  ? [En ligne]. http://www.elfe-france.fr/index.php/fr/. Consulté le 20 avril 2017.

**Background** : MASTER 2 Degree in Public Health

**Supervisor cONTACT** : MADAME CHARLES MARIE-ALINE

**EMAIL** : MARIE-ALINE.CHARLES@INSERM.FR

**Phone** : 01.45.59.51.05

#

**Signature of the Team Manager**

AVIS FAVORABLE

SIGNATURE


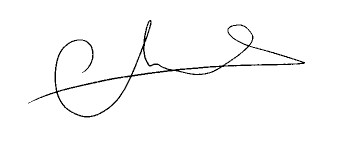

Supplement: S1 Protocol — (DOCX) [file pmed.1002871.s003.docx]
