# Supplementary material for: An association between maternal weight change in the year before pregnancy and infant birth weight: ELFE, a French national birth cohort study
Source: PLoS Med. 2019 Aug 20;16(8):e1002871. doi: 10.1371/journal.pmed.1002871 (PMC6701747; doi:10.1371/journal.pmed.1002871)
Supplement: S1 Questionnaire — (PDF) [file pmed.1002871.s004.pdf]

23 / 05 / 2016

MATERNITY UNIT SURVEY
Administered in 2011
MEDICAL FILE DATA

Data collected face-to-face
Questionnaire for of-age mothers of children born at 33 weeks or more
(single or twin births)

Greyed-out questions must absolutely be completed

UPD **Updates (constructed variables and corrected variables) are included at the end of the chapter, indicated by this symbol.**

CONTENTS

GENERAL INFORMATION 2

PREGNANCIES 3

PRIOR PATHOLOGIES 5

INFECTIOUS PATHOLOGIES, SCREENING, TREATMENT 6

OTHER PATHOLOGIES AND COMPLICATIONS 11

PRENATAL IMAGING AND DIAGNOSIS 13

DELIVERY 16

CHILD 19

TRANSFER 24

TRANSFERRED CHILD: PATHOLOGIES 29

TRANSFERRED CHILD: DIET, OTHER INFORMATION 36

GENERAL INFORMATION

<DMNIE>
Medical data ID: |__|__|__|__|__|

DM
DM questionnaire available
0 Unavailable
1 Available

<DMUSERNAME>
Interviewer ID: ___________________________

*DMCREATION
Date created .... |__|__| |__|__| |__|__|

*TRMOTABSQ
Reason for absence of questionnaire: _____________________________

PREGNANCIES

NGROSS
Total number of pregnancies (not including current pregnancy) |__|__|

If NGROSS>0:
NACC
Number of births (not including current birth)............. |__|__|

NMORTNE
Number of stillbirths: |__|__|

NDNEONAT
Number of neonatal deaths (between 0 and 27 days) |__|__|

NPREMA
Number of children born prematurely (<37 weeks) |__|__|

NHYPOT
Number of children born small for gestational age |__|__|

GMULT
Number of multiple pregnancies |__|__|

ANTCESAR
Previous caesarian births:
0 No
1 Yes
9 Doesn't know

***DREGLES**
Date of last period (dd/mm/yy) ... |__|__| |__|__| |__|__|

***DCONCEPC**
UPD
Start date of pregnancy (corrected) |__|__| |__|__| |__|__|

JUMEAUX
Expecting twins?
0 No
1 Yes
9 Doesn't know

BIRTH ORDER
Order of twin .... |__|__|

RANGALEA
Order of twin (generated randomly, enabling random selection of one of the twins) |__|__|

TYPJUM

Is the twin pregnancy:

1 Monochorionic/monoamniotic
2 Monochorionic/diamniotic
3 Dichorionic
4 Other
9 Doesn't know

(Question asked starting from wave 2)

PRIOR PATHOLOGIES

HTA
High blood pressure
0 No
1 Chronic HBP
2 HBP during a prior pregnancy only

DIABETE
Diabetes
0 No
1 Type 1 (insulin dependent)
2 Type 2 (non insulin dependent)
3 Pregnancy-related diabetes (prior pregnancies)

AUTPATHO
Other chronic or severe pathology/pathologies or handicap(s)
0 No
1 Yes
9 Doesn't know

If AUTPATHO=1
***AUTPATHOP**
Specify: ________________________

INFECTIOUS DISEASES, SCREENING, TREATMENT

Blood analysis made between 3rd and 8th month (if several, choose first)

HEMOGL
Haemoglobin (g/100 ml) (value between 5 and 25) I__I__I ,I__I

HEMAT
Haematocrit % (value between 20 and 50) I__I__I ,I__I

*DBS
Date of analysis (dd/mm/yy) I__I__I I__I__I I__I__I
⇨ *See end of chapter for corrected variable and constructed variable: date of analysis in amenorrhoea days*

TOXO
Serological status relative to toxoplasmosis (result of last test made during pregnancy):
1 Lack of antibodies (seronegative woman)
2 Presence of IgMs (alone or together with specific IgGs)
3 Presence of specific IgGs (immunised woman)
4 Test not carried out due to known immunisation (positive test before pregnancy)
5 Test not carried out (unknown reason)
6 No information on test realisation or result

If TOXO≠1
TRTTOXO
Treatment against toxoplasmosis
0 No
1 Spiramycin (Rovamycine®)
2 Pyrimethamine / Sulfamides (Malocide® + Adiazine® or Fansidar®)
3 Both
4 Treatment carried out but drug(s) non-specified
9 Not documented

If TRTTOXO>0
DATETOXO
Treatment start date known?
0 No
1 Yes
9 Not documented

If DATETOXO=1
*DTRTTOXO
Specify: I__I__I I__I__I I__I__I
⇨ *See end of chapter for corrected variable and constructed variable: treatment start date in amenorrhoea days*

SEROCONV
Seroconversion dating:
1 Documented seroconversion (positivation of serology of toxoplasmosis during the pregnancy)
2 Suspected infection acquired during the pregnancy
9 Not documented

If SEROCONV=1

*DEXNTOXO
Date of last negative examination (dd/mm/yy) |__|__||__|__||__|__|
⇨ *See end of chapter for corrected variable and constructed variable: date of last negative examination in amenorrhoea days*

*DEXPTOXO
Date of last positive examination(dd/mm/yy) |__|__||__|__||__|__|
⇨ *See end of chapter for corrected variable and constructed variable: date of last positive examination in amenorrhoea days*

If SEROCONV=2
***DPTOXOSI**
Date of first positive examination (seroconversion undocumented) (dd/mm/yy) |__|__||__|__||__|__|
⇨ *See end of chapter for corrected variable and constructed variable: date of first positive examination in amenorrhoea days (seroconversion undocumented)*

CMV
Serological status regarding CMV (result of first test carried out during pregnancy)
1 Lack of antibodies (seronegative woman)
2 Presence of IgMs (alone or together with specific IgGs)
3 Presence of specific IgGs (immunised woman)
4 Test not carried out
5 No information

AGHBSG
Testing the mother for the HBs antigen during current pregnancy
1 HBs Ag negative
2 HBs Ag positive
3 Test not carried out due to known HBs Ag before pregnancy
4 Test not carried out because woman vaccinated
5 Test not carried out, other reason (e.g. unmonitored pregnancy)
6 No information on test realisation or result

INT: IMPORTANT: THIS IS THE HBs Ag, NOT THE ANTI-HBs Ab

If AGHBSG≠1
AGHBSPN
Testing for HBs Ag after birth:
1 HBs Ag negative
2 HBs Ag positive
3 Test not carried out owing to known HBs Ag before pregnancy
4 Test not carried out because woman vaccinated
5 No information on test realisation or result
9 Not documented

If AGHBSPN≠1
IGHBENF
Was/were the child/children given anti-HBs immunoglobulins? (IM-LFB)
0 No
1 Yes
2 Unknown
9 Not documented

If IGHBENF=1
***DIGHBENF**
Date of injection (dd/mm/yy) |__|__| |__|__| |__|__|
⇨ *See end of chapter for corrected variable and constructed variable: number of days between child birth date and corrected injection date*

If AGHBSPN≠1
VACCVHB
Was/were the child/children vaccinated for HB (first dose)? (GenHevac B, Engenrix B10, HBvac Pro5)
0 No
1 Yes
2 Unknown
9 Not documented

If VACCVHB=1
***DVACCVHB**
Date of injection (dd/mm/yy) |__|__| |__|__| |__|__|
⇨ *See end of chapter for corrected variable and constructed variable: number of days between child birth date and vaccination date*

DEPSYPHG
Screening for syphilis (TPHA and VRDL) during pregnancy?
1 Yes, once
2 Yes, several times
3 No examination
4 No information on realisation or results of tests

If DEPSYPHG<3
DEPIS
Positive?
0 No
1 Yes
9 Doesn't know

If DEPIS=1
TRTEXT
Treated with Extencilline (injectable)?

0 No
1 Yes
2 Unknown
9 Doesn't know

If DEPIS=1
SDEPSYPHG
Pregnancy period in weeks of amenorrhoea I__I__I

***DBSC**
UPD **Date of six-month examination (corrected)** I__I__I I__I__I
I__I__I

DBSJR
UPD **(Constructed variable) Date of six-month examination in amenorrhoea days:** I__I__I
Corrected date of six-month examination – conception date + 14

***DTRTTOXOC**
UPD **Start date of toxoplasmosis treatment (corrected)** I__I__I I__I__I I__I__I

DTRTTOXOJR
UPD **(Constructed variable) Start date of treatment in amenorrhoea days** I__I__I
Start date of treatment – conception date + 14

***DEXNTOXOC**
UPD **Date of last toxo examination (documented seroconversion) (corrected)** I__I__I I__I__I I__I__I

DEXNTOXOJR
UPD **(Constructed variable) Date of last negative examination in amenorrhoea days**
I__I__I
Date of last negative examination – conception date + 14
***DEXPTOXOC**
UPD **Date of first toxo + examination (documented seroconversion) (corrected)**I__I__I I__I__I I__I__I

DEXPTOXOJR
UPD **(Constructed variable) Date of first toxo positive examination in amenorrhoea days** I__I__I
Date of first positive examination – conception date + 14

DPTOXOSIJR
UPD **(Constructed variable) Date of first toxo positive examination in amenorrhoea days (undocumented seroconversion)** I__I__I
Date of first positive examination – conception date + 14

***DIGHBENFC**
UPD **Date child injected with anti-HBs immunoglobulins (corrected)** I__I__I I__I__I I__I__I

DIGHBENFJR
UPD **(Constructed variable) Number of days between date of birth and corrected date of anti-HBs immunoglobulins injection** I__I__I

***DVACCVHBC**
UPD **Date of HB vaccination (corrected)** I__I__I I__I__I I__I__I

DVACCVHBJR

UPD **(Constructed variable) Number of days between date of birth and date of HB vaccination**

|__|__|

OTHER PATHOLOGIES AND COMPLICATIONS

MAP
Threat of premature delivery (repeated and painful uterine contractions and/or cervical modifications)
0 No
1 Yes, having required hospitalisation
2 Yes, with outpatient treatment only (medicine and/or rest)

RUPTMEMB
Premature rupture of membranes (at least 12 hours before start of labour)
0 No
1 Yes
9 Doesn't know
(Question duplicated for twins)

If RUPTMEMB=1

***DRUPTMEMB**
Date of rupture (dd/mm) |__|__| |__|__|
⇨ *See end of chapter for corrected variable and constructed variable: Date of membrane rupture in amenorrhoea days*

(Question duplicated for twins)

HEMORR
Haemorrhage in second or third trimester
0 No
1 Placenta praevia
2 Retroplacental haematoma
3 Other

HTAG
High blood pressure developed during pregnancy (systolic ≥ 140 mmHg or diastolic ≥ 90 mmHg)
0 No
1 Yes, with proteinuria (≥ 0.3 g/l or per 24h)
2 Yes, without proteinuria

If HTAG>0

***DHTAG**
Date at diagnosis (dd/mm) |__|__| |__|__|
⇨ *See end of chapter for corrected variable and constructed variable: Date at HBP diagnosis in amenorrhoea days*

If HTAG>0
TRTHTAG
Treatment?
0 No

1 Yes
9 Not documented

DIABGEST
Gestational diabetes
0 No
1 Yes
9 Doesn't know

If DIABGEST=1
DIABGESTP
Treatment?
1 Insulin
2 Diet only
3 Not documented

ANOPOI
Suspected foetal weight anomaly during pregnancy
0 No anomaly
1 Intra-uterine growth restriction (IUGR)
2 Macrosomia
(Question duplicated for twins)

CORTIC
Prenatal administration of corticosteroids for foetal maturation
0 No
1 Yes
9 Doesn't know

If CORTIC=1
***DCORTIC**
Date of first injection |__|__| |__|__|
⇨ *See end of chapter for corrected variable and constructed variable: Date of first injection in amenorrhoea days*

NBCORTIC
Number of treatments (1 treatment= several injections) |__|

CORTICP
Corticosteroid administered
1 Betamethasone
2 Dexamethasone
3 Other
9 Doesn't know

***DRUPTMEMBC**
UPD **Date of membrane rupture (corrected)** |__|__| |__|__|

DRUPTMEMBJR
UPD **(Constructed variable) Date of membrane rupture in amenorrhoea days** |__|__|
Date of membrane rupture (corrected) – conception date + 14

***DHTAGC**
UPD **Date at HBP diagnosis (corrected)** |__|__||__|__|

DHTAGJR
UPD **(Constructed variable) Date at HBP diagnosis in amenorrhoea days** |__|__|
Date at HBP diagnosis (corrected) – conception date + 14

***DCORTICC**
UPD **Date of first injection (corrected)** |__|__||__|__|

DCORTICJR
UPD **(Constructed variable) Date of first injection in amenorrhoea days** |__|__|
Date of first corticosteroid injection (corrected) – conception date + 14

PRENATAL IMAGING AND DIAGNOSIS

(Duplicated for twins)

First-trimester ultrasound

ECHO1SEM
Gestational age in amenorrhoea weeks |__|__|

ECHO1JR
Gestational age: days in addition to number of weeks |__|__|

ECHO1CC
Measurement of cranio-caudal (CC) length (mm) |__|__|__|

ECHO1LF
Length of femur (LF) (mm) |__|__|__|

ECHO1DAT
Transverse abdominal diameter (TAD) (mm) |__|__|__|

ECHO1CA
If TAD unavailable, abdominal circumference (mm) |__|__|__|

ECHO1BIP
Biparietal diameter (BPD) (mm) |__|__|__|

Second-trimester ultrasound

ECHO2SEM
Gestational age in amenorrhoea weeks |__|__|

ECHO2SEMJ
Gestational age: days in addition to number of weeks |__|__|

ECHO2LF
Length of femur (LF) (mm) |__|__|__|

ECHO2DAT
Transverse abdominal diameter (TAD) (mm) |__|__|__|

ECHO2CA
If TAD unavailable, abdominal circumference (mm) |__|__|__|

ECHO2BIP
Biparietal diameter (BPD) (mm) |__|__|__|

Third-trimester ultrasound

ECHO3SEM
Gestational age in amenorrhoea weeks |__|__|

ECHO3SEMJ
Gestational age: days in addition to number of weeks

ECHO3LF
Length of femur (LF) (mm) |__|__|__|

ECHO3DAT
Transverse abdominal diameter (TAD) (mm) |__|__|__|

ECHO3CA
If TAD unavailable, abdominal circumference (mm) |__|__|__|

ECHO3BIP
Biparietal diameter (BPD) (mm) |__|__|__|

Have any of the following examinations been made?
AMNIO
Amniocentesis
0 No
1 Yes
2 Unknown
9 Doesn't know

BIOPSY
Trophoblast biopsy
0 No
1 Yes
2 Unknown
9 Doesn't know

PRELEVSF

Foetal blood sample
0 No
1 Yes
2 Unknown
9 Doesn't know

If AMNIO=1 or BIOPSIE=1 or PRELEVSF=1 *(one of the examinations has been made)*
MOTEXAM
For what reason?
1 Maternal age alone
2 Nuchal translucency
3 Another ultrasound warning sign
4 Blood test
5 Integrated risk (nuchal translucency + blood test)
6 Another reason, code and specify
9 Doesn't know

If MOTEXAM=6
***MOTEXAMP Specify ____________________________**

If AMNIO=1 or BIOPSIE=1 or PRELEVSF=1 *(one of the examinations has been made)*
Has the examination led to:

ANOCONG
The diagnosis of a congenital anomaly
0 No
1 Yes
2 Unknown
9 Doesn't know

TOXOCONG
The diagnosis of a congenital toxoplasmosis
0 No
1 Yes
2 Unknown
9 Doesn't know

CMVCONG
The diagnosis of a CMV congenital infection
0 No
1 Yes
2 Unknown
9 Doesn't know

DELIVERY

Gestational age at delivery

AGEGESTS
Weeks I__I__I

AGEGESTJ
Days I__I

PRESENT
Type of birth
1 Cephalic
2 Breech
3 Other
9 Doesn't know
(Question duplicated for twins)

DEBTRAV
Start of labour
1 Spontaneous labour
2 Induced *(including cervical ripening alone)*
3 Caesarean before start of labour

If DEBTRAV>1
Reasons

MOTTRAV_1
Intra-uterine growth restriction
0 No
1 Yes
9 Doesn't know

MOTTRAV_2
Severe foetal distress (IUGR anomaly, reduced foetal movements, anomaly with umbilical or cerebral dopplers)
0 No
1 Yes
9 Doesn't know

MOTTRAV_3
Suspected chorion amnionitis
0 No
1 Yes
9 Doesn't know

MOTTRAV_4
Maternal reason
0 No

1 Yes
9 Doesn't know

MOTTRAV_5
No medical reason
0 No
1 Yes
9 Doesn't know

MOTTRAV_6
Other
0 No
1 Yes
9 Doesn't know

INT: 2 POSSIBLE REASONS

If MOTTRAV_4=1
***MOTTRAVM**
Specify _____________________

If MOTTRAV_6=1
***MOTTRAVP**
Specify _____________________

ANALG
Analgesia
0 None
1 Epidural
2 Spinal anaesthesia
3 Combined epidural/spinal anaesthesia (or sequential)
4 General anaesthetic
5 Other

INT: NOT INCLUDING POST-DELIVERY ANAESTHETICS, SAY THE HIGHEST

ANALG=1
ANALGP
Specify _______________________

TYPACC
Delivery
1 Spontaneous vaginal
2 Forceps, spatulas, vacuum
3 Caesarean
9 Doesn't know
(Question duplicated for twins)

If TYPACC=3
TYPCES
Was the caesarean:

1 Planned
2 Carried out as an emergency
9 Doesn't know
(Question duplicated for twins)

If TYPACC >1 (caesarean or vaginal using instruments)
MOTCES
Reason
1 IUGR or lactate anomaly
2 Another foetal indication
3 Another maternal indication
4 Other
9 Doesn't know

INT: 2 POSSIBLE REASONS

(Question duplicated for twins)

If MOTCES=4
***MOTCESP**
Specify ___________________________
(Question duplicated for twins)

EPISIO
Episiotomy
0 No
1 Yes
9 Doesn't know

POIPLAC
Weight of placenta (grammes) I__I__I__I__I
(Question duplicated for twins)

CHILD

(Duplicated for twins)

REA
Reanimation in delivery room
0 No
1 Yes
9 Doesn't know

If REA=1
REAP
Reanimation technique
1 Bag-mask ventilation
2 NeoPuff ventilation
3 Nasal CPP
4 Intubation
9 Doesn't know

PRLVCORD
Umbilical cord sample
0 No
1 Yes
9 Doesn't know

If PRLVCORD=1
PHCORD
pH |__|, |__|__|

LACCORD
Lactates (mmol/l) |__|__|, |__|__|

PRLVPERI
Peripheral blood sample
0 No
1 Yes
9 Doesn't know

If PRLVPERI=1
TPSPERI
At what hour of life? |__|__|

PHPERI
pH |__|, |__|__|

LACPERI
Lactates (mmol/l) |__|__|, |__|__|

APGAR1M
Apgar at 1 minute |__|__|

APGAR5M
Apgar at 5 minutes I__I__I

***SEXEC1**
(Select the SEX variable from the FCC maternity unit questionnaire)
UPD
Sex (corrected)
1 Male
2 Female
9 Doesn't know

POIENF
Weight (grammes) I__I__I__I__I

TAIENF
Size (cm) I__I__I
INT: IF THE MEASUREMENT WAS NOT MADE ON BIRTH, GIVE THE VALUE OF A MEASUREMENT MADE BEFORE LEAVING THE MATERNITY UNIT *(text added in wave 3)*

PC
Cranial perimeter (cm) I__I__I
INT: IF THE MEASUREMENT WAS NOT MADE ON BIRTH, GIVE THE VALUE OF A MEASUREMENT MADE BEFORE LEAVING THE MATERNITY UNIT *(text added in wave 3)*

Girl
GENIC1
UPD
Anomaly with external genital organs (corrected)
0 No
1 Yes
9 Doesn't know

If GENI=1
***GENIPC1**
UPD
Specify (corrected) ____________________________

Boy
TESTICC1
UPD
At least one undescended testicle (corrected)
0 No
1 Yes
9 Doesn't know

HYPOSPC1
UPD
Hypospadias (corrected)
0 No
1 Yes

9 Doesn't know

MICROPC1
UPD
Micropenis (corrected)
0 No
1 Yes
9 Doesn't know

GENIAUTC1
UPD
Other malformation of external genital organs (corrected)
0 No
1 Yes
9 Doesn't know

If GENIAUT=1
***GENIAUTPC1**
UPD
Specify (corrected) ________________________________

ANOAUT
Other congenital anomaly
0 No
1 Yes
9 Doesn't know

If ANOAUT=1
***ANOAUTP Specify** ______________________________

DMATER
Maternity death (if live birth)
0 No
1 Yes
9 Doesn't know

PHOTOTH
Phototherapy
0 No
1 Yes
9 Doesn't know

If PHOTOTH=1
PHOTOP
Type of phototherapy
1 Intensive
2 Conventional
3 Not documented

ANTIBIO

IV antibiotherapy
0 No
1 Yes
9 Doesn't know

GAVAGE
Tube feeding
0 No
1 Yes
9 Doesn't know

TESTAUD
Hearing screening
0 No test
1 Otoacoustic emissions (OAEs)
2 Automatic auditory evoked potentials (AEPs)
3 Not documented

If TESTAUD=1 or 2
RESTEST
Result:
1 Normal
2 Bilateral abnormal
3 Unilateral abnormal
9 Not documented

POISORTCONNU
Was the child's weight known at the end of the survey period?
0 No
1 Yes
9 Doesn't know

INT: WAIT UNTIL THE LAST DAY OF THE SURVEY TO ANSWER THIS QUESTION NEGATIVELY IN ORDER TO DOCUMENT THE HIGHEST NUMBER OF EXIT WEIGHTS POSSIBLE

(Question added in wave 2)
If POISORTCONNU=1
POISORT
Exit weight of child (grammes) |__|__|__|__|

*DSORTENF
Maternity unit exit date (child) (dd/mm/yy) |__|__| |__|__| |__|__|
⇨ *See end of chapter for corrected variable and constructed variable: Number of days between the child's date of birth and corrected date of leaving maternity unit (child)*

*DSORTM
Maternity unit exit date (mother) (dd/mm/yy) |__|__| |__|__| |__|__|
⇨ *See end of chapter for corrected variable and constructed variable: Number of days between the child's date of birth and corrected date of leaving maternity unit (mother)*

HOSPM

Particular hospitalisation or transfer of mother
0 No
1 Yes
9 Doesn't know

If HOSPM=1
HOSPMP
Specify:
1 In reanimation or intensive care for over 24 hours
2 In another unit for maternal medical reason
3 To be with the child
9 Doesn't know

***DSORTENFC**
UPD **Maternity unit exit date (child) (corrected)** |__|__| |__|__| |__|__|

DSORTENFJR
UPD **(Constructed variable) Number of days between the child's date of birth and maternity unit exit date (corrected) (child)** |__|__|

***DSORTMC**
UPD **Maternity unit exit date (mother) (corrected)** |__|__| |__|__| |__|__|

DSORTMJR
UPD **(Constructed variable) Number of days between the child's date of birth and maternity unit exit date (corrected) (mother)** |__|__|

TRANSFER

(Duplicated for twins)

TRANS
Transfer of child
0 No
1 Yes
9 Doesn't know

Reason for transfer

MOTTRAN_1
Prematurity or hypotrophy
0 No
1 Yes
9 Doesn't know

MOTTRAN_2
Respiratory distress
0 No
1 Yes
9 Doesn't know

MOTTRAN_3
Suspected infection
0 No
1 Yes
9 Doesn't know

MOTTRAN_4
Congenital anomaly
0 No
1 Yes
9 Doesn't know

MOTTRAN_5
Other
0 No
1 Yes
9 Doesn't know

INT: 2 POSSIBLE REASONS

If MOTTRAN_5=1
*MOTTRANP
Other reason, specify ________________________________

ETTRANS
Transfer:
1 In same establishment

2 To another establishment
9 Doesn't know

If ETTRANS=1 (transfer in same establishment)
TRMMET
To which unit?
1 Reanimation or intensive care
2 Neonatal
3 Neonatal unit in the maternity unit (including "kangaroo" mother-child units)
4 Other
9 Doesn't know

If TRMMET=4
***TRMMETP Other unit, specify: ______________________________**

If ETTRANS=1 (transfer to another establishment)
TRAUTET
To which unit?
1 Reanimation or intensive care
2 Neonatal
3 Other
9 Doesn't know

If TRAUTET=3
***TRAUTETP**
Other unit, specify ______________________________

➔ Child 2 if twins, or end of questionnaire

TRNBTRANS
Number of transfers |__|__|

*TRD1TRANS
Date of arrival at first transfer location |__|__| |__|__| |__|__|
⇨ *See end of chapter for the corrected variable and constructed variable: number of days between the child's date of birth and the date of arrival at the first transfer location (corrected)*

TRT1SERV
Unit:
1 Reanimation
2 Neonatal
3 Unit in maternity unit
4 Other
9 Doesn't know

If TRT1SERV=9
***TRT1SERVP**
Other unit, specify ___________________________

TRT1NIV
Level:
1 Level 1
2 Level 2A

3 Level 2B
4 Level 3
9 Doesn't know

***TRT1VILLE**
Town ___________________________

***TRDDERTRANS**
Date of arrival at last transfer location |__|__| |__|__| |__|__|
➪ *See end of chapter for the corrected variable and constructed variable: number of days between the child's date of birth and the date of arrival at the last transfer location (corrected)*

TRTDSERV
Unit:
1 Reanimation
2 Neonatal
3 Unit in maternity unit
4 Other
9 Doesn't know

***TRTDSERVP**
Other unit, specify: ___________________________

TRTDNIV
Level:
1 Level 1
2 Level 2A
3 Level 2B
4 Level 3
9 Doesn't know

***TRTDVILLE**
Town: ___________________________

***TRDSORTIE**
Departure date from last transfer location |__|__| |__|__| |__|__|
➪ *See end of chapter for the corrected variable and constructed variable: number of days between the child's date of birth and the date of departure from the last transfer location (corrected)*

TRDESTI
Departure from last transfer location to:
1 Home
2 Maternity unit
3 Nursery
4 Death
5 Other
9 Doesn't know

If TRDESTI=5
***TRDESTIP**

Other destination, specify ___________________________

If TRDESTI=4

***TRDDECES**

Date of death |__|__| |__|__| |__|__|

***TRPQDECES**

Cause of death ___________________________

RECAP OF HOSPITAL LOCATIONS

TRREANIM

Reanimation unit:

0 No

1 Yes

9 Doesn't know

TRDURREA

Total length of stay (in days): |__|__|

TRNEONAT

Neonatal unit:

0 No

1 Yes

9 Doesn't know

TRNEONMATER

Neonatal unit in maternity unit:

0 No

1 Yes

9 Doesn't know

TRCHIRURG

Surgery:

0 No

1 Yes

9 Doesn't know

TRAUTSERV

Other specialised unit:

0 No

1 Yes

9 Doesn't know

***TRAUTSERVP**

Other specialised unit, specify ___________________________

TRDECES

Death:

0 No

1 Yes

9 Doesn't know

***TRD1TRANSC**
UPD **Date of arrival at first transfer location (corrected)** I__I__I I__I__I I__I__I

TRD1TRANSJR
UPD **(Constructed variable) Number of days between the child's date of birth and the date of arrival at the first transfer location (corrected)** I__I__I

***TRDDERTRANSC**
UPD **Date of arrival at last transfer location (corrected)** I__I__I I__I__I I__I__I

TRDDERTRANSJR
UPD **(Constructed variable) Number of days between the child's date of birth and the date of arrival at the last transfer location (corrected)** I__I__I

***TRDSORTIEC**
UPD **Departure date from last transfer location (corrected)** I__I__I I__I__I I__I__I

TRDSORTIEJR
UPD **(Constructed variable) Number of days between the child's date of birth and the departure date from the last transfer location (corrected)**
I__I__I

TRANSFERRED CHILD: PATHOLOGIES

TRPATHORESP
Respiratory pathology
0 No
1 Yes
9 Doesn't know

If TRPATHORESP=1
TRDETRESP
Respiratory distress of less than 48 hours:
0 No
1 Yes
9 Doesn't know

TRMBHYAL
Hyaline membrane disease
0 No
1 Yes
9 Doesn't know

TRINHALMEC
Meconium Aspiration Syndrome:
0 No
1 Yes
9 Doesn't know

***TRAUTRESP**
Other respiratory pathology, specify ___________________________

TRRADTHOR
Chest X-rays:
0 No
1 Yes
9 Doesn't know

If TRRADTHOR=1
TRNBRADTHOR
Number of chest X-rays: |__|__|

TRTDMTHOR
Chest CT scans:
0 No
1 Yes
9 Doesn't know

If TRTDMTHOR=1
TRNBTDMTHOR
Number of chest CT scans: |__|__|

TRINTUB
Intubation:
0 No
1 Yes
9 Doesn't know

TRDURVENTIL
Total number of days of ventilation (mechanical, infant flow, CPP): I__I__I

Specify any details:
TRDURVENTMECA
Number of days of mechanical ventilation I__I__I

TRDURVENTIF
Number of days of infant flow I__I__I

TRDURVENTPPC
Number of days of CPP I__I__I

TROXYGEN
Oxygen therapy
0 No
1 Yes
9 Doesn't know

If TROXYGEN=1
TRDUROXYGEN
Number of days of oxygen therapy I__I__I

***TRDOXYGEN**
Date of definitive end of oxygen therapy I__I__I I__I__I I__I__I
⇨ *See end of chapter for the corrected variable and constructed variable: number of days between the child's date of birth and the date of the definitive end of oxygen therapy (corrected)*

TRINFPRECOC
Early infection (72 hours of life or less)
0 No
1 Yes
9 Not documented

If TRINFPRECOC=1
TRIPGERME
Germ identified?
0 No
1 Yes
9 Not documented

If TRIPGERME=1
TRIPQLGERM
Which germ?

1 Streptococcus B
2 E. Coli
3 Other
9 Doesn't know

If TRIPQLGERM=3
TRIPQLGERMP
Other germ, specify ______________________

TRIPMENING
Meningeal infection?
0 No
1 Yes
9 Doesn't know

TRINFTARD
Late infection (after 72 hours of life)
0 No
1 Yes
9 Doesn't know

If TRINFTARD=1
TRITGERME
Germ identified?
0 No
1 Yes
9 Doesn't know

If TRITGERME=1
TRITQLGERM
Specify ______________________

TRANTIBIOIV
IV antibiotherapy
0 No
1 Yes
9 Doesn't know

If TRANTIBIOIV=1
TRDURANTIBIO
Length in days: I__I__I

TRICTERE
Icteurs (treated with phototherapy or exchange transfusion)
0 No
1 Yes
9 Doesn't know

If TRICTERE=1
TRTXBILIRUB
Maximum rate of bilirubin (micromol/L) I__I__I

TRAGICTERE
Rate attained at which age (in days, 00 if under 24 hours)**?** I__I__I

TRPATHODIG
Digestive pathology
0 No
1 Yes
9 Doesn't know

If TRPATHODIG=1
***TRPATHODIGP**
Specify ________________________

TRRADABDO
Abdomen X-rays without preparation:
0 No
1 Yes
9 Doesn't know

If TRRADABDO=1
TRNBRADABDO
Specify the number: I__I__I

TRTDMABDO
Abdomen CT scan
0 No
1 Yes
9 Doesn't know

If TRTDMABDO=1
TRNBTDMABDO
Specify the number: I__I__I

TRANOCHROM
Chromosomal malformation or anomaly
0 No
1 Yes
9 Doesn't know

If TRANOCHROM=1
TRCARYOTYP
Karyotype required
0 No
1 Yes
9 Doesn't know

If TRCARYOTYP=1
TRRESCARYO
Result:
1 Pending
2 Normal
3 Abnormal

9 Doesn't know

If TRRESCARYO=1
***TRRESCARYOP**
Describe the anomaly in detail ___________________________________

TRNEURO
Neurological anomaly or pathology
0 No
1 Yes
9 Doesn't know

If TRNEURO=1
TRENCEPHALO
Encephalopathy
0 No
1 Yes
9 Doesn't know

If TRENCEPHALO=1
TRENCEPHALOP
Stage (1, 2 or 3): |__|

TRECHOTF
Cranial ultrasound
0 No
1 Yes
9 Doesn't know

TRHEMORRIV
Intra-ventricular haemorrhage
0 No
1 IVH I
2 IVH II
3 IVH III
4 IVH IV
9 Doesn't know

TRLEUCOM
Leukomalacia
0 No
1 Hyperechogenicity > 14 days
2 Cavitary
9 Doesn't know

TRDILATVENT
Ventricular dilatation
0 No
1 Yes
9 Doesn't know

TRTDMCER

Brain CT scan
0 No
1 Yes
9 Doesn't know

If TRTDMCER=1
TRNBTDMCER
Specify the number: |__|__|

TRRESTDMCER
Result:
0 Normal
1 Abnormal
9 Doesn't know

If TRTDMCER=1
***TRTDMCERP**
Specify the anomaly: ______________________________________

TRIRMCER
Brain MRIs
0 No
1 Yes
9 Doesn't know

If TRIRMCER=1
TRNBIRMCER
Specify the number: |__|__|

TRRESIRMCER
Result:
1 Normal
2 Abnormal
9 Doesn't know

If TRRESIRMCER=2
***TRRESIRMCERP Specify the anomaly** _____________________________

***TRPATHOOBST**
Specify __________________________

TRRADAUT
X-ray performed
0 No
1 Yes
9 Doesn't know

If TRRADAUT=1
TRRADLOC Specify location _____________________________

TRTDMAUT
CT scan performed

0 No
1 Yes
9 Doesn't know

If TRTDMAUT=1
TRTDMLOC **Specify location** ______________________________

TRIRMAUT
MRI performed
0 No
1 Yes
9 Doesn't know

If TRIRMAUT=1
TRIRMLOC **Specify location** ______________________________

*TRDOXYGENC
UPD **Date of definitive end of oxygen therapy (corrected)** |__|__| |__|__| |__|__|

TRDOXYGENJR
UPD **Number of days between the child's date of birth and the definitive end of oxygen therapy (corrected)** |__|__|

TRANSFERRED CHILD: FEEDING AND OTHER INFORMATION

TRJDEBALIM
Start of feeding, specify which day of life: I__I__I

TRCATHOMB
Umbilical venous catheter
0 No
1 Yes
9 Doesn't know

TRCATHCENT
Central catheter
0 No
1 Yes
9 Doesn't know

If TRCATHCENT=1
TRDURCATHC
Number of days: I__I__I

TRRADPOSEC
Inspection X-rays for fitting catheter:
0 No
1 Yes
9 Doesn't know

If TRRADPOSEC=1
TRNBRADPOSEC
Number of X-rays: I__I__I

TRGAVAGE
Gastric force-feeding
0 No
1 Yes
9 Doesn't know

If TRGAVAGE=1
TRDURGAVAGE
Number of days: I__I__I

TRLAITMAT
Mother's milk:
0 No
1 Yes
9 Doesn't know

TRLAITPA
Initial milk:
0 No

1 Yes
9 Doesn't know

TRLAITAUT
Other:
0 No
1 Yes
9 Doesn't know

TRTESTAUD
Hearing screening
0 No test
1 Otoacoustic emissions (OAEs)
2 Automatic auditory evoked potentials (AEPs)
9 Not documented

If TRTESTAUD=1 or 2
TRRESTEST
Result:
1 Normal
2 Bilateral abnormal
3 Unilateral abnormal
9 Doesn't know

TRMEREENF
Mother present with her child:
1 Every day or almost
2 A few days a week
3 Once a week
4 Less than once a week
9 Doesn't know

TRPOISORT
Child's weight at departure (grammes): |__|__|__|__|

TRPCSORT
Cranial perimeter at departure (cm): |__|__|__|__|

END
