# Supplementary material for: An association between maternal weight change in the year before pregnancy and infant birth weight: ELFE, a French national birth cohort study
Source: PLoS Med. 2019 Aug 20;16(8):e1002871. doi: 10.1371/journal.pmed.1002871 (PMC6701747; doi:10.1371/journal.pmed.1002871)
Supplement: S2 Questionnaire — (PDF) [file pmed.1002871.s005.pdf]

MATERNITY UNIT SURVEY
Administered in 2011
INTERVIEW WITH THE MOTHER

Ined
Inserm
EFS
InVS
Insee
DGS
DGPR
Drees
Cnaf

Data collected face to face
Questionnaire for of-age mothers born at 33 weeks or more
(single or twin births)

Greyed-out questions must absolutely be completed

UPD **Updates (constructed variables and corrected variables) are included at the end of the chapter, indicated by this symbol.**

CONTENTS

GENERAL INFORMATION 2

SOCIO-DEMOGRAPHIC SITUATION 4

EDUCATION AND OCCUPATIONAL SITUATION 8

SITUATION BEFORE YOUR PREGNANCY 11

YOUR PREGNANCY 12

PSYCHOLOGICAL ASPECTS 14

IMAGING EXAMINATIONS: SCANNER, MRI, X-RAY, ULTRASOUND 16

SCREENING 23

NUTRITION, TOBACCO AND ALCOHOL 25

HOLIDAYS – UV EXPOSURE 28

DELIVERY AND CHILD 30

GENERAL INFORMATION

<FAFNIE>
Bar code: |__|__|__|__|__|

FAF
FTF questionnaire available
0 Unavailable
1 Available

<FAFUSERNAME>
Interviewer ID ___________________________

*FAFCREATION
Questionnaire creation date |__|__||__|__||__|__|

*LIEUNAISSENF
Maternity unit in which child was born ______________________

*COMNAISM2
Maternity unit municipality ___________________________

*DEPNAISM2
Postcode of the maternity unit |__|__|__|__|__|

NAISS
Birth
0 Single
1 Multiple

*DNAISSE
Date of birth of child (first born if twin pregnancy): |__|__||__|__||__|__|

ENFTRANS
Child transferred:
If ENFTRANS=0 then DNAISSM (page 2)
0 No
1 Yes

If ENFTRANS=1 and NAISS=1
QLENFTRAN
Which child is concerned?
1 The first born
2 The second born
3 Both
9 No answer

TRPREV

Were you informed before the birth that your baby/babies may be transferred?

0 No
1 Yes
9 No answer

TREXPL

Was the reason for the transfer clearly explained to you?

1 Yes, absolutely
2 Yes, somewhat
3 No, not really
4 No, not at all
9 No answer

MVUBB

Were you able to see your baby/babies before the transfer?

0 No
1 Yes
9 No answer

PVUBB

Was the father of the baby/babies able to see him/her/them?

0 No
1 Yes, saw him/her/them
2 Yes, saw him/her/them and accompanied him/her/them
9 No answer

MERETR

Were you able to transfer to the building or establishment to which your baby/babies was/were transferred?

0 No
1 Yes
9 No answer

SOCIO-DEMOGRAPHIC SITUATION

***DNAISSM**
What is your date of birth? (dd/mm/yy) |__|__||__|__||__|__|

ANAISSM
(Constructed variable) Date of birth of the mother |__|__|__|__|

⇨ *See end of chapter for the constructed variable: age of the mother at birth*

LIEUNAISM
Were you born in France or in another country?
1 In France
2 In another country

If LIEUNAISM=1
CPCONNUM
Do you know the postcode of your town of birth?
0 No
1 Yes
9 No answer

If CPCONNUM=1
***DEPNAISM**
What is that postcode? |__|__|__|__|__|

***COMNAISM**
What municipality were you born in? _______________________

If CPCONNUM=0
***DEPM**
What department were you born in? |__|__|

***COMM**
What municipality were you born in? _______________________

***COMCLAIRM**
Municipality of the mother _______________________

If LIEUNAISM=2
***PAYSNAISM**
In which country? _______________________

⇨ *See end of chapter for the constructed variable*

NATIOM
What is your nationality?
1 French by birth (including by reintegration)
2 French by acquisition (naturalisation, marriage, declaration, or choice when coming of age)
3 Foreign
4 Stateless

INT: DO NOT SUGGEST

ETATMAT
What is your marital status?
1 Married or remarried (including legally separated)
2 Civil partnership
3 Divorced
4 Single
5 Widow

INT: PROPOSE ANSWERS. IMPORTANT: THE STATUS OF A WOMAN SAYING SHE IS IN A COMMON-LAW UNION NECESSARILY CORRESPONDS TO ONE OF THE FIVE PROPOSED.

COUPLE
Are you currently in a couple?
0 No
1 Yes

If ETATMAT≠1
RECONU
Has the father recognised your child?
0 No
1 Yes
9 No answer

INT: PROPOSE ANSWERS

***DNAISSP**
What is the date of birth of the child's father? |__|__||__|__||__|__|
⇨ *See end of chapter for the constructed variable: date of birth of child*

ANAISSP
Year of birth of father |__|__|__|__|
⇨ *See end of chapter for the constructed variable: age of father at birth*

LIEUNAISP
Was he born in France or in another country?
1 In France
2 In another country

If LIEUNAISP=1
CPCONNUP
Do you know the postcode of the municipality he was born in?
0 No
1 Yes
9 No answer

If CPCONNUP=1
***DEPNAISP**
What is that postcode? |__|__|__|__|__|

*COMNAISP
What municipality was he born in? _______________________

If CPCONNUP=0
*DEPP
What department was he born in? |__|__|

*COMP
What municipality was he born in? _______________________

*COMCLAIRP
Municipality of the father: _______________________

If LIEUNAISP=2
*PAYSNAISP
In which country? _______________________

⇨ *See end of chapter for the constructed variable*

NATIOP
What is his nationality?
1 French by birth (including by reintegration)
2 French by acquisition (naturalisation, marriage, declaration, or choice when coming of age)
3 Foreign
4 Doesn't know

AGEM
UPD **(Constructed variable) Age of the mother at the date of birth of the ELFE child** |__|__|, |__|

AGEP
UPD **(Constructed variable) Age of the father at the date of birth of the ELFE child** |__|__|, |__|

PAYS2NAISM
UPD **(Constructed variable) In which country?**
0 France
1 European Union and EEE
2 Turkey
3 Morocco
4 Algeria
5 Tunisia
6 French-speaking Sub-Saharan Africa
7 Other Sub-Saharan Africa
8 Other country
9 Not stated

⇨ *See end of chapter for the constructed variable*

PAYS2NAISP

UPD **(Constructed variable) In which country?**

0 France
1 European Union and EEE
2 Turkey
3 Morocco
4 Algeria
5 Tunisia
6 French-speaking Sub-Saharan Africa
7 Other Sub-Saharan Africa
8 Other country
9 Not stated

⇨ See end of chapter for the constructed variable

JNAISSEALEA

UPD **(Constructed variable) Date of birth of child (ELFE theoretical days, generated randomly)?**

|__|__|

PAYS5NAISM

UPD **(Constructed variable) In which country?**

1 European Union
2 Turkey
3 Morocco
4 Algeria
5 Tunisia
6 French-speaking Sub-Saharan Africa
7 Other Sub-Saharan Africa
8 Eastern/Central Europe
9 Asia
10 South/Central America
11 Other
12 Not stated

PAYS5NAISP

UPD **(Constructed variable) In which country?**

1 European Union
2 Turkey
3 Morocco
4 Algeria
5 Tunisia
6 French-speaking Sub-Saharan Africa
7 Other Sub-Saharan Africa
8 Eastern/Central Europe
9 Asia
10 South/Central America
11 Other
12 Not stated

EDUCATION AND OCCUPATIONAL SITUATION

NIVET

What is your educational level?

1 Primary school
2 Middle school (age 11-15)
3 Classes preparing a vocational training certificate or school-leaving diploma
4 General high-school classes for 15-18 year-olds
5 Technical high-school classes for 15-18 year-olds
6 Occupational high-school classes for 15-18 year-olds
7 Higher education (university, etc.)
8 You never attended school

*PROFESSP

What is your current or most recent occupation? ______________________

INT: INDICATE OCCUPATION IN DETAIL IF NO OCCUPATION, WRITE "NONE" AND NOTE DOWN NOTHING FOR **PROFESS**

PROFMAUCUN

No occupation: |__|

PROFESS

In which of these categories would you put your occupation?

1 Farmer
2 Self-employed: business, trade or crafts
3 Manager, professional and higher-level intellectual occupation
4 Intermediate occupation (teacher, nurse, technician, supervisor, etc.)
5 Clerical or sales worker
6 Manual worker
7 No occupation
9 Unable to categorise occupation

CSP1M

Constructed variable: Social category completed from 2-month questionnaire for mothers having taken part in two-month and whose social category was missing or incorrectly entered in the maternity unit

1 Farmer
2 Self-employed: business, trade or crafts
3 Manager, professional and higher-level intellectual occupation
4 Intermediate occupation (teacher, nurse, technician, supervisor, etc.)
5 Clerical or sales worker
6 Manual worker
7 No occupation
9 Unable to categorise occupation

SITUG

When you became pregnant

1 You had a job
2 You were a homemaker
3 You were a student or in training

4 You were unemployed
5 You were on parental leave
6 You were retired
7 You were in another situation

INT: PROPOSE ALL ANSWERS

If SITUG=1

*DARRETT
When did you stop working, i.e. without returning to your job before the delivery?
⇨ *See end of chapter for the corrected variable and constructed variable: date stopped work in amenorrhoea days*

***PROFESSCP**
What is the current or most recent occupation of the child's father?

INT: INDICATE OCCUPATION IN DETAIL IF NO OCCUPATION, WRITE "NONE" AND NOTE DOWN NOTHING FOR THE NEXT QUESTION

PROFAUCUN
No occupation: |__|

PROFESSC
In which of these categories would you put that occupation?
1 Farmer
2 Self-employed: business, trade or crafts
3 Manager, professional and higher-level intellectual occupation
4 Intermediate occupation (teacher, nurse, technician, supervisor, etc.)
5 Clerical or sales worker
6 Manual worker
7 No occupation
9 Unable to categorise occupation

CSP1P
Constructed variable: Social category completed from 2-month questionnaire for mothers having taken part in two-month and whose social category was missing or incorrectly entered in the maternity unit
1 Farmer
2 Self-employed: business, trade or crafts
3 Manager, professional and higher-level intellectual occupation
4 Intermediate occupation (teacher, nurse, technician, supervisor, etc.)
5 Clerical or sales worker
6 Manual worker
7 No occupation
9 Unable to categorise occupation

EMPLOIC
Currently, the father of your child
1 Has a job
2 Is a homemaker
3 Is a student or in training

4 Is unemployed
5 Is on parental leave
6 Is retired
7 Is in another situation

If EMPLOIC=7
***EMPLOICP Specify clearly:** ________________________
(Question deleted from wave 2 onwards)

SS1
Which social security system manages your healthcare costs?
1 Universal healthcare coverage (CMU)
2 The general system or another system connected to the general system: civil servants, state workers, agents of local authorities, authorised healthcare occupations, artists, religious occupations, Alsace-Moselle, disabled ex-servicemen, disabled adult allowance beneficiaries, disability pensions
3 The agricultural social security system: MSA, AMEXA, ASA, GAMEX, AAEXA - (including those of Alsace-Moselle)
4 The system for independent professionals: RSI, AMPI, RAM: (self-employed, business, trade and crafts, manufacturers, liberal professions, doctors charging their own fees)
5 Another system: EDF/GDF, SNCF, Mines, RATP, military careers, students, border-dwellers
6 State Medical Aid (AME)
7 The system of a family member of friend insured in another country
8 Doesn't know which system
9 No system (no coverage)

CMCOMP
Do you have supplementary healthcare coverage (mutual insurance, private insurance, personal risk, including supplementary CMU) that reimburses your healthcare costs in addition to the social security system?
1 Yes
2 No
9 Doesn't know

If CMCOMP=1
CMU
Is it CMU?
0 No
1 Yes
9 Doesn't know

***DARRETTC**
UPD **Date stopped work before delivery (corrected)** |__|__||__|__||__|__|

DARRETTJR
UPD **(Constructed variable) Date stopped work in amenorrhoea days:** |__|__|
date stopped work – corrected conception date + 14

Situation before your pregnancy

"I'm going to ask you a few questions about your health before and during your pregnancy."

GANT
Had you ever been pregnant before this pregnancy (including abortions or miscarriages)?
0 No
1 Yes

If GANT=1
ENFGANT
Had you already had children?
0 No
1 Yes
9 Doesn't know

If ENFGANT=1
NBGANT
How many (not including the child just born)? |__|__|

If GANT=1
IVG
During your previous pregnancies, did you have one or more abortions, whether by surgery or drug-induced?
0 No
1 Yes
9 Doesn't know

TRTSTER
For this pregnancy, did you have infertility treatment?
0 No
1 Yes

YOUR PREGNANCY

REACG

When you found out you were pregnant:

1 You were happy that the pregnancy was occurring now
2 You would have liked it to happen earlier
3 You would have liked it to happen later
4 You would have preferred not to be pregnant

DECLGQD

When did you declare your pregnancy?

0 Undeclared pregnancy
1 In the first trimester
2 In the second trimester
3 In the third trimester

If DECLGQD≠0

DECLGQI

Who declared your pregnancy?

1 A gynaecologist or obstetrician in a private practice or as part of a mother-and-child-protection programme
2 A gynaecologist or obstetrician in a maternity unit
3 A GP
4 A midwife in a maternity unit
5 A midwife in a private practice
6 Other
9 Doesn't know

If DECLGQI=6

***DECLGQIP Specify** ________________________

(Question deleted from wave 2 onwards)

SUIVIG

Throughout your pregnancy, you were monitored...

1 Mainly by a gynaecologist or obstetrician (or several)
2 Mainly by a GP (or several)
3 Mainly by a midwife (or several)
4 By several of these professionals
5 By none of these professionals
6 By no-one

NVISPREN

How many prenatal visits did you have in all? |__|__|

NVPMAT

Of these visits, how many were at a maternity unit or with a person belonging to the medical team of a maternity unit? |__|__|

HOSPG
Were you hospitalised during this pregnancy (including same-day hospitalisations)?
0 No
1 Yes

If HOSPG=1
DURHOSPG
What was the total length of these hospital stays? (In days) |__|__|__|

ENT4MOIS
Did you have a four-month interview?
1 Yes
2 No
9 Doesn't know

PREPNAIS
Did you attend birth classes during this pregnancy?
0 No
1 Yes

If PREPNAIS=1
NPREPNAIS
How many? |__|__|

If NAISS=1 (twin pregnancy)
TYPJUM
Are your twins monozygotic or dizygotic?
1 Monozygotic
2 Dizygotic
3 Doesn't know

If NAISS=1 (twin pregnancy)
TYPGGEM
Was your pregnancy monochorionic or dichorionic?
1 Monochorionic
2 Dichorionic
3 Doesn't know

If TYPGGEM=1 (monochorionic twin pregnancy)
GMONOCH
And was it monoamniotic or diamniotic?
1 Monoamniotic
2 Diamniotic
3 Doesn't know

PSYCHOLOGICAL ASPECTS

"I am now going to ask you a few questions about your experience of the pregnancy."

DIFFPSY
During the pregnancy, did you have any persistent psychological difficulties?
0 No
1 Yes

If DIFFPSY=1
CONSLPSY
During your pregnancy, did you see a psychiatrist, a psychiatrist or a psychotherapist concerning these difficulties?
0 No
1 Yes
9 Doesn't know

If DIFFPSY=1
MEDPSY
Did you take any medicine for these difficulties during your pregnancy?
0 No
1 Yes
9 Doesn't know

If MEDPSY=1
MEDANX
Specifically, did you take any medicine for anxiety during your pregnancy?
0 No, not at all
1 Yes, once
2 Yes, several times
9 Doesn't know

If MEDPSY=1
MEDDEP
Medicine for depression?
0 No, not at all
1 Yes, once
2 Yes, several times
9 Doesn't know

If MEDPSY=1
MEDHUM
Medicine to alter your mood?
0 No, not at all
1 Yes, once
2 Yes, several times
9 Doesn't know

DEPGR
And during a previous pregnancy, were you depressed or very anxious?
0 No
1 Yes
9 Doesn't know

If DEPGR=1
How long did that last?
DEPMOI In months: |__|__|
Or **DEPSEM In weeks:** |__|__|

INT: IF SEVERAL PREGNANCIES, THE LONGEST PERIOD

IMAGING EXAMINATIONS: SCANNER, MRI, X-RAY, ULTRASOUND

"We are now going to talk about your medical examinations during your pregnancy."

NECHO
How many ultrasounds did you have in all (whatever the location)? |__|__|

SCANNER
Did you have any scans during your pregnancy?
0 No
1 Yes

If SCANNER=1
In which trimester and for what reason?

Entire body:

SCORT
Trimester |__|

SCORM
Reason
1 Pelvimetry
2 Trauma
3 Chronic illness
4 Other
5 Doesn't know

Head:

STETT
Trimester |__|
STETM
Reason
1 Pelvimetry
2 Trauma
3 Chronic illness
4 Other
5 Doesn't know

Chest:

STHOT
Trimester |__|

STHOM
Reason
1 Pelvimetry
2 Trauma
3 Chronic illness

4 Other
5 Doesn't know

Stomach (abdomen):

SABDOT
Trimester I__I

SABDOM
Reason
1 Pelvimetry
2 Trauma
3 Chronic illness
4 Other
5 Doesn't know

Pelvis or hips:

SBASST
Trimester I__I

SBASSM
Reason
1 Pelvimetry
2 Trauma
3 Chronic illness
4 Other
5 Doesn't know

Limb (wrist, hand, foot, ankle, leg, arm, shoulder):

SMEMBT
Trimester I__I
SMEMBM
Reason
1 Pelvimetry
2 Trauma
3 Chronic illness
4 Other
5 Doesn't know

Kidneys, bladder:

SREINT
Trimester I__I

SREINM
Reason
1 Pelvimetry
2 Trauma
3 Chronic illness

4 Other
5 Doesn't know

INT: FILL IN FOR PART OF BODY CONCERNED

IRM
Did you have an MRI during your pregnancy?
0 No
1 Yes

If IRM=1
In which trimester and for what reason?

Entire body:

ICORT
Trimester I__I

ICORM
Reason
1 Pelvimetry
2 Trauma
3 Chronic illness
4 Other
5 Doesn't know

Head:

ITETT
Trimester I__I

ITETM
Reason
1 Pelvimetry
2 Trauma
3 Chronic illness
4 Other
5 Doesn't know

Chest:

ITHOT
Trimester I__I

ITHOM
Reason
1 Pelvimetry
2 Trauma
3 Chronic illness
4 Other
5 Doesn't know

Stomach (abdomen):

IABDOT
Trimester |__|

IABDOM
Reason
1 Pelvimetry
2 Trauma
3 Chronic illness
4 Other
5 Doesn't know

Pelvis or hips:

IBASST
Trimester |__|

IBASSM
Reason
1 Pelvimetry
2 Trauma
3 Chronic illness
4 Other
5 Doesn't know

Limb (wrist, hand, foot, ankle, leg, arm, shoulder):

IMEMBT
Trimester |__|
IMEMBS
Reason
1 Pelvimetry
2 Trauma
3 Chronic illness
4 Other
5 Doesn't know

Kidneys, bladder:

IREINT
Trimester |__|

IREINM
Reason
1 Pelvimetry
2 Trauma
3 Chronic illness
4 Other
5 Doesn't know

INT: FILL IN FOR PART OF BODY CONCERNED

RADIO
Did you have an X-ray during your pregnancy?
0 No
1 Yes

If RADIO=1
In which trimester and for what reason?

Entire body:

RCORT
Trimester I__I

RCORM
Reason
1 Pelvimetry
2 Trauma
3 Chronic illness
4 Other
5 Doesn't know

Head:

RTETT
Trimester I__I

RTETM
Reason
1 Pelvimetry
2 Trauma
3 Chronic illness
4 Other
5 Doesn't know

Chest:

RTHOT
Trimester I__I

RTHOM
Reason
1 Pelvimetry
2 Trauma
3 Chronic illness
4 Other
5 Doesn't know

Stomach (abdomen):

RABDOT
Trimester I__I

RABDOM
Reason
1 Pelvimetry
2 Trauma
3 Chronic illness
4 Other
5 Doesn't know

Pelvis or hips:

RBASST
Trimester I__I

RBASSM
Reason
1 Pelvimetry
2 Trauma
3 Chronic illness
4 Other
5 Doesn't know

Limb (wrist, hand, foot, ankle, leg, arm, shoulder):

RMEMBT
Trimester I__I
RMEMBM
Reason
1 Pelvimetry
2 Trauma
3 Chronic illness
4 Other
5 Doesn't know

Kidneys, bladder:

RREINT
Trimester I__I

RREINM
Reason
1 Pelvimetry
2 Trauma
3 Chronic illness
4 Other
5 Doesn't know

INT: FILL IN FOR PART OF BODY CONCERNED

SCREENING

"Now let's talk about any illnesses you were tested for during your pregnancy."

DEPVIHG
Were you screened for AIDS (HIV) during your pregnancy?
1 Yes
2 No
9 Doesn't know

If DEPVIHG=2
NONVIH
Why?
1 Not proposed
2 You refused
3 Last recent examination
4 Other reason
5 Doesn't know

If NONVIH=4
***NONVIHP**
Other reason, specify ________________________
(Question deleted from wave 2 onwards)

CONTOXO
During your pregnancy, did you receive any preventive advice to avoid contracting toxoplasmosis?
1 Yes
2 No, because immunised
3 No, with no further precisions
4 Doesn't know

DEPDG
Were you screened for gestational diabetes?
0 No
1 Yes, once
2 Yes, several times
3 Doesn't know

If DEPDG=1 or 2 (screened for diabetes)
Was it done on the basis of:
TDEPDG_1
Fasting glycaemia
0 No
1 Yes
9 Doesn't know

TDEPDG_2
Post-prandial glycaemia (after a meal)
0 No
1 Yes

TDEPDG_3

Glucose level test

0 No

1 Yes

TDEPDG_4

Doesn't know

0 No

1 Yes

INT: SEVERAL ANSWERS POSSIBLE

NUTRITION, TOBACCO AND ALCOHOL

ACIDEFOL
Did you take folic acid (vitamin B9) before and/or during your pregnancy (to prevent nervous system problems)?
1 Yes
2 No
9 Doesn't know

If ACIDEFOL=1
Indicate the periods during which you took it:

PERIODAF_1
1 to 3 months before the pregnancy
0 No
1 Yes
9 Doesn't know

PERIODAF_2
In the first two months of the pregnancy
0 No
1 Yes

PERIODAF_3
Between the second and sixth month of the pregnancy
0 No
1 Yes

PERIODAF_4
After the sixth month of the pregnancy
0 No
1 Yes

INT: SEVERAL ANSWERS POSSIBLE

POIMAVTG
How much did you weigh before this pregnancy? (in kg) |__|__|__|

POIMFING
How much did you weigh at the end of this pregnancy? (in kg) |__|__|__|

TAIM
How tall are you? (in cm) |__|__|__|

TABAVTG
Before your pregnancy, had you smoked for at least one year?
0 No
1 Yes
9 Doesn't know

If TABAVTG=1
DBTTABA
At what age did you start smoking? |__|__|

If TABAVTG=1
ARRTABA
Have you ever stopped smoking?
0 No
1 Yes
9 Not documented

If ARRTABA=1
AGARRTAB
How old were you when you last stopped? |__|__|

If TABAVTG=1
NBCIGAG
On average, for the entire period in which you smoked, before stopping or cutting down, how much did you smoke? |__|__|

UTCIGAG
Unit
1 Pack(s)
2 Cigarette(s)
9 Not documented

FRCIGAG
Per
1 Day
2 Week
3 Month
9 Not documented

TABAG
Did you smoke during your pregnancy, even just from time to time?
0 No
1 Yes
9 Doesn't know

If TABAG=1
TABA3G
Was that the case during the third trimester of your pregnancy?
0 No
1 Yes
9 Not documented

If TABA3G=1
NBCIG3G
How much on average? |__|__|

UTCIG3G
Unit
1 Pack(s)
2 Cigarette(s)
9 Not documented

FRCIG3G
Per
1 Day
2 Week
3 Month
9 Not documented

If TABA3G=1 *(Filter provided for: TABA3G=0, programming error)*
MSTOPTAB
At what month of your pregnancy did you stop smoking? |__|__|

EXPTABD
During your pregnancy, were you exposed to the tobacco smoke of other people in your household?
0 Never or almost never
1 Less than an hour a day
2 1 to 2 hours a day
3 2 to 5 hours a day
4 Over five hours a day

EXPTABLF
During your pregnancy, were you exposed to the tobacco smoke of other people in any other closed environment (workplace, leisure venue, bar, restaurant, etc.)?
0 Never or almost never
1 Less than an hour a day
2 1 to 2 hours a day
3 2 to 5 hours a day
4 Over 5 hours a day

If EXPTABLF=4
EXPTABLFP
How many hours a day? |__|__|

FQALCOOL
During your pregnancy, how often did you consume alcoholic drinks (beer, cider, wine, aperitifs, etc.)?
0 Never
1 Once a month or less frequently, or at special occasions such as parties
2 2 to 4 times a month
3 2 to 3 times a week
4 4 times a week or more but not every day
5 Every day
6 Only before you knew you were pregnant
7 Prefers not to answer

HOLIDAYS – UV EXPOSURE

HOLIDAYS

Did you take any holidays outside your habitual residence during your pregnancy, for four days or more? *If VACANCES = 0, then PEREACC (page 20)*

0 No

1 Yes

If VACANCES=1

NBVAC

How many times? |__|__|

Detail on each holiday:

VPAYS1

Which country did you visit?

1 France

2 Another country

9 Not documented

If VPAYS1=2

VPAYSE1

Which country? ________________

VDPT1

Department (if France): ____________________

VVILLE1

Town(s): _________________________

Type of place:

VTYPE1_1

Beach

0 No

1 Yes

VTYPE1_2

Mountain

0 No

1 Yes

VTYPE1_3

Other

0 No

1 Yes

INT: SEVERAL ANSWERS POSSIBLE

VDEXACT1
Do you know the exact dates of your stay?
0 No
1 Yes

If VDEXACT1=1
VDDEPART1
Departure date: |__|__||__|__||__|__|

VDRETOUR1
Return date: |__|__||__|__||__|__|

If VDEXACT1=0
VMDEPART1
Month of departure: ___________________

VMRETOUR1
Month of return: ___________________

DUREESEJ1
Length of stay (in days): |__|__|

VEXPOSOL1
Average exposure to sun (hours per day): |__|__|

If VEXPOSOL1>0
EXPOVOL1
Did you voluntarily expose yourself to the sun?
0 No
1 Yes
9 Not documented

If VEXPOSOL1>0
VCREMSOL1
Use of sunscreen:
0 No
1 Yes
9 Not documented

INT: REPEAT THIS PART UP TO 10 TIMES IN THE EVENT OF OTHER HOLIDAYS

DELIVERY AND CHILD

"I'd like to finish with a few questions about your baby/babies."

PEREACC
Was the father present at the delivery?
0 No
1 Yes

How is/are your baby/babies currently being fed?
ALIMENF_1
Mother's milk only
0 No
1 Yes

ALIMENF_2
Initial milk only
0 No
1 Yes

ALIMENF_3
Combined (mother's milk and initial milk)
0 No
1 Yes

ALIMENF_4
Doesn't know
0 No
1 Yes

ALIMENF_5
Other
0 No
1 Yes

⇨ *See end of chapter for corrected variables*

If ALIMENF_5=1
***ALIMENFP**
Specify ________________________
(Question deleted from wave 2 onwards)

If ALIMENF_2=1 or ALIMENF_5=1
NONALLAIT
Is it because you didn't want to breastfeed your child/children?
0 No
1 Yes
9 Not documented

CHALIM
What motivated your feeding choice for your child/children?
1 The child's health or well-being
2 Practical reasons
3 Financial reasons
4 Establishing a relationship with the child
5 Normal choice, customary in life environment, was fed herself that way
6 Medical prescriptions/contraindications
7 Other

SUIVIENF
Have you planned to have someone monitor the health of your children after leaving?
0 No
1 Yes

If SUIVIENF=1
SUIVIENFQ
Who?
1 Your GP
2 Another GP
3 A paediatrician in a private practice
4 Mother-and-child protection centre (PMI)
5 Other
9 Not documented

If SUIVIENFQ=5
***SUIVIENFP**
Specify ____________________

How is/are your baby/babies currently being fed?
ALIMENFC_1
UPD **Mother's milk only (corrected)**
0 No
1 Yes

ALIMENFC_2 **(corrected)**
UPD **Initial milk only (corrected)**
0 No
1 Yes

ALIMENFC_3
UPD **Combined (mother's milk and initial milk) (corrected)**
0 No
1 Yes

ALIMENFC_4
UPD **Doesn't know (corrected)**
0 No
1 Yes

ALIMENFC_5

UPD **Other (corrected)**

0 No

1 Yes

"Thank you for taking part."

END
