# Supplementary material for: An association between maternal weight change in the year before pregnancy and infant birth weight: ELFE, a French national birth cohort study
Source: PLoS Med. 2019 Aug 20;16(8):e1002871. doi: 10.1371/journal.pmed.1002871 (PMC6701747; doi:10.1371/journal.pmed.1002871)
Supplement: S3 Questionnaire — (DOCX) [file pmed.1002871.s006.docx]

Maternal Food questionnaire – Weight variations

Have you ever gone on a diet to lose weight? Yes No

If yes, it was:

In the year before your pregnancy Yes No

During your pregnancy Yes No

Did your weight vary in the year before your pregnancy?

(you can check several boxes if your weight went up or down several times)

Gain > 5 kg

Gain between 2 and 5 kg

Stable weight

Loss between 2 and 5 kg

Loss > 5 kg
