# Supplementary material for: An association between maternal weight change in the year before pregnancy and infant birth weight: ELFE, a French national birth cohort study
Source: PLoS Med. 2019 Aug 20;16(8):e1002871. doi: 10.1371/journal.pmed.1002871 (PMC6701747; doi:10.1371/journal.pmed.1002871)
Supplement: S1 Table — *Fully conditional specification method. **Percent interval of missing data for the different z-scores of fetal growth in the second and third trimester. (DOCX) [file pmed.1002871.s008.docx]

| Variable | Type of variable | Model used to predict missing data* | Missing value  % |
| --- | --- | --- | --- |
| Mother’s place of birth  Health insurance coverage  Smoking before pregnancy  Smoking during pregnancy  Living with a partner  Parity  Number of ultrasounds  Restrictive diet before pregnancy  Restrictive diet during pregnancy  Gestational diabetes  Hypertension  Psychological difficulties during pregnancy  Hospitalization during pregnancy  Premature rupture of membranes  Maternal medical history that can influence weight variations  Maternal medical history that could influence fetal growth  Women with child ≤ 2 years  Child’s sex | Binary  Binary  Binary  Binary  Binary  Binary  Binary  Binary  Binary  Binary  Binary  Binary  Binary  Binary  Binary  Binary  Binary (yes/no)  Binary | Logistic regression  Logistic regression  Logistic regression  Logistic regression  Logistic regression  Logistic regression  Logistic regression  Logistic regression  Logistic regression  Logistic regression  Logistic regression  Logistic regression  Logistic regression  Logistic regression  Logistic regression  Logistic regression  Logistic regression  Logistic regression | 0.1%  0.5%  0.5%  0.8%  0.6%  0.3%  3%  12.4%  15.3%  4.3%  2.1%  0.6%  0.8%  2.7%  6.1%  6.1%  0%  0.1% |
| Maternal age  Maternal weight  Maternal height  Body mass index  Gestational weight gain  Gestational age at birth  Birth weight  Birth length  Head circumference at birth Ultrasonography data second trimester (Z-scores)  Ultrasonography data third trimester (Z-scores) | Continuous  Continuous  Continuous  Continuous  Continuous  Continuous  Continuous  Continuous  Continuous  Continuous  Continuous | Linear regression  Linear regression  Linear regression  Linear regression  Linear regression  Linear regression  Linear regression  Linear regression  Linear regression  Linear regression  Linear regression | 0.02%  1.3%  0.03%  1.3%  2%  0.8%  1.4%  9%  9.7%  25-69%**  20-69%** |
| Weight variations before pregnancy  Activity status  Maternal level of education  Marital status  Socio-professional category  Complementary health assurance  Medical history of diabetes  Medical history of hypertension  Pregnancy caregiver  Prenatal consultations  Consumption of alcohol  Maternity size strata  Period of inclusion  Mode of birth | Categorical (3 categories)  Categorical (3 categories)  Categorical (4 categories)  Categorical (3 categories)  Categorical (5 categories)  Categorical (3 categories)  Categorical (4 categories)  Categorical (3 categories)  Categorical (4 categories)  Ordinal (3 categories)  Categorical (4 categories)  Categorical (5 categories)  Categorical (4 categories)  Categorical (3 categories) | Multinomial regression  Multinomial regression  Multinomial regression  Multinomial regression  Multinomial regression  Multinomial regression  Multinomial regression  Multinomial regression  Multinomial regression  Multinomial regression  Multinomial regression  Multinomial regression  Multinomial regression  Multinomial regression | 13.2%  2.4%  0.01%  2%  0%  2.1%  2.1%  1.4%  0.9%  1.8%  0.7%  0%  0%  1.9% |
