# Supplementary material for: An association between maternal weight change in the year before pregnancy and infant birth weight: ELFE, a French national birth cohort study
Source: PLoS Med. 2019 Aug 20;16(8):e1002871. doi: 10.1371/journal.pmed.1002871 (PMC6701747; doi:10.1371/journal.pmed.1002871)
Supplement: S2 Table — Data are percent (n) or mean ± SD. *By chi-squared test comparing the 3 groups of weight variation or ANOVA for continuous variables. (DOCX) [file pmed.1002871.s009.docx]

| **Variables** | **N** | **Weight loss (N= 1415)** | **Stable weight (N=11210)** | **Weight Gain (N=1668)** | **P-value*** |
| --- | --- | --- | --- | --- | --- |
| **Maternal age (years)** | 14289 | 30.2 ± 5.0 | 30.8 ± 4.9 | 29.8 ± 5.4 | <0.01 |
| **Place of birth (France)** | 14287 | 92.1 (1301) | 90.4 (10128) | 79.3 (1323) | <0.01 |
| **Maternal education** | 14293 |  |  |  |  |
| At most lower secondary school |  | 8.8 (124) | 6.0 (668) | 15.3 (256) | <0.01 |
| Upper secondary |  | 40.6 (574) | 31.6 (3539) | 45.1 (753) |  |
| Post-secondary |  | 23.2 (328) | 23.8 (2665) | 19.4 (324) |  |
| Tertiary |  | 27.5 (389) | 38.7 (4338) | 20.1 (335) |  |
| **Activity status** | 14027 |  |  |  |  |
| Employed or student |  | 79.2 (1094) | 84.2 (9292) | 67.6 (1091) | <0.01 |
| Women staying at home |  | 20.8 (288) | 15.8 (1740) | 32.4 (522) |  |
| **Social health insurance coverage** | 14249 |  |  |  |  |
| Precarious situations |  | 7.6 (108) | 5.5 (618) | 15.7 (260) | <0.01 |
| Regular |  | 92.4 (1306) | 94.5 (10556) | 84.3 (1401) |  |
| **Living with a partner (Yes)** | 14227 | 94.9 (1336) | 95.9 (10697) | 91.5 (1521) | <0.01 |
| **Smoking before pregnancy (Yes)** | 14262 | **49.8 (702)** | 42.6 (4765) | 40.8 (678) | <0.01 |
| **Smoking during pregancy (Yes)** | 14219 | **24.7 (347)** | 19.3 (2151) | 20.9 (347) | <0.01 |
| **Smoking behavior** | 14154 |  |  |  |  |
| No smoking before and during pregnancy |  | 49.9 (697) | 57.3 (6363) | 58.9 (969) | <0.01 |
| Smoking before pregnancy and not after |  | **25.6 (358)** | 23.7 (2632) | 20.9 (343) |  |
| Smoking before and during pregnancy |  | 24.4 (341) | 19.1 (2118) | 20.2 (333) |  |
| **Pregnancy** |  |  |  |  |  |
| **Primiparous (Yes)** | 14273 | 36.3 (512) | 45.2 (5065) | 43 (717) | <0.01 |
| **Pregnancy caregiver** | 14206 |  |  |  |  |
| Gynaecologist |  | 65.9 (926) | 66.8 (7440) | 63.5 (1053) | 0,13 |
| Midwife |  | 11.5 (161) | 11.9 (1325) | 12.9 (214) |  |
| General pratictioner or no one |  | 6.1 (86) | 5.4 (598) | 6.6 (110) |  |
| Several professionals |  | 16.6 (233) | 16 (1779) | 16.9 (281) |  |
| **Restrictive diet before pregnancy (Yes)** | 14127 | 57.2 (802) | 10.5 (1159) | 23.9 (391) | <0.01 |
| **Body mass index (BMI) kg/m²** | 14293 | 25.5 ± 5.2 | 22.7 ± 4.2 | 26.2 ± 5.4 | <0.01 |
| **Gestational weight gain (kg)** | 14203 | 14.47 ± 6.77 | 13.0 ± 5.02 | 12.9 ± 6.1 | <0.01 |
