# Supplementary material for: An association between maternal weight change in the year before pregnancy and infant birth weight: ELFE, a French national birth cohort study
Source: PLoS Med. 2019 Aug 20;16(8):e1002871. doi: 10.1371/journal.pmed.1002871 (PMC6701747; doi:10.1371/journal.pmed.1002871)
Supplement: S3 Table — Adjusted model: linear regression adjusted for maternal education level, maternal age, smoking before and during pregnancy, place of birth, parity, health insurance coverage, active status, and pre-pregnancy BMI. aMinimum–maximum number of women in each category of weight variation before pregnancy depending on imputed tables: weight loss, 910–944; stable weight, 10,177–10,192; and weight gain, 923–956. bMinimum–maximum number of women in each category of weight variation before pregnancy depending on imputed tables: weight loss, 690–703; stable weight, 2,543–2,564; and weight gain, 1,072–1,097. (DOCX) [file pmed.1002871.s010.docx]

|  | **Gestational weight gain** | |
| --- | --- | --- |
| Weight variations | **Unadjusted model** | **Adjusted model** |
|  | **β (CI%95)** | **β (CI%95)** |
| **BMI < 25 kg/m²** |  |  |
| ***Weight change before pregnancy*** |  |  |
| Weight loss ^a^ | **2.25 [1.84 ;2.66]** | **2.20 [1.79 ;2.61]** |
| Stable weight ^a^ | *0 [Ref]* | *0 [Ref]* |
| Weight gain ^a^ | **0.92 [0.59 ;1.24]** | **1.12 [0.79 ;1.45]** |
| **BMI ≥ 25 kg/m²** |  |  |
| ***Weight change before pregnancy*** |  |  |
| Weight loss ^b^ | **2.28 [1.66 ;2.89]** | **2.76 [2.21 ;3.32]** |
| Stable weight ^b^ | *0 [Ref]* | *0 [Ref]* |
| Weigth gain ^b^ | **0.84 [0.31 ;1.37]** | **1.61 [1.12 ;2.11]** |
